# The influence of negative affective states on the neurophysiological correlates of prospective memory

**DOI:** 10.1186/s41235-026-00749-3

**Published:** 2026-08-09

**Authors:** Francesco Pupillo, Jasna Martinovic, Louise H. Phillips, Katharina Schnitzspahn, Alexandra Hering

**Affiliations:** 1 School of Psychology, University of Aberdeen, Aberdeen, UK; 2 Tilburg School of Social and Behavioural Sciences, Tilburg University, Tilburg, Netherlands; 3 School of Philosophy, Psychology and Language Sciences, University of Edinburgh, Edinburgh, UK; 4 Faculty of Psychology and Educational Sciences, University of Geneva, Geneva, Switzerland

**Keywords:** Prospective memory, Negative affective state, ERP, Emotion-cognition interactions

## Abstract

Prospective memory (PM), the ability to remember to perform an intended action in the future, is influenced by affective states. We tested two opposing theoretical accounts regarding the effects of negative affect on cognitive tasks using behavioral and neural data to better understand the mechanisms underlying emotional effects on PM. We presented negative or neutral film clips to participants prior to them completing an event-based PM task while their electrophysiological activity was recorded with electroencephalography (EEG). Results revealed significant interactions between affective state and trial type (PM vs. non-PM trials) for the N300 and FN400 components, indicating that the relationship between affective state and ERP amplitude varied across trial types. No comparable interactions were observed for the P3b or prospective positivity components. In addition, higher levels of intrusive thoughts were associated with poorer PM performance and reduced N300 amplitudes. The overall pattern is consistent with the possibility that negative affective states influence early PM processes, potentially via increased task-unrelated thoughts and reduced allocation of attentional resources. These findings provide tentative support for capacity-based accounts and highlight the need for further research to clarify the reliability and magnitude of these effects.

**Supplementary Information:**

The online version contains supplementary material available at https://doi.org/10.1186/s41235-026-00749-3.

## Significant statement

In everyday life, people often need to remember to carry out intended actions at the right moment, such as taking medication when a specific cue appears, responding to an alert at work, or delivering a message when encountering someone. These prospective memory demands frequently occur under conditions of negative mood, yet we still have limited understanding of how experiencing emotions influences the underlying cognitive and neural mechanisms that support remembering future intentions. The present study addresses this real-world problem by combining basic cognitive neuroscience methods with questions motivated by everyday challenges. Using electrophysiological measures, we examined how negative affect alters the neural processes involved in the realization of intended actions, such as detecting and recognizing cues that signal when the actions should be performed. By focusing on neurophysiological responses, the study moves beyond observable behavior and reveals changes in cognitive processing that may precede overt performance failures. This work advances fundamental knowledge about how emotion interacts with attention and memory, while remaining directly relevant to real-world situations in which prospective memory is critical. The findings show that neural processes may vulnerable to negative affective states and intrusive thoughts, helping to understand why remembering intentions can become more difficult under negative emotional states and providing a foundation for developing strategies to support prospective memory in everyday and applied settings.

**Supplementary Information:**

The online version contains supplementary material available at https://doi.org/10.1186/s41235-026-00749-3.

## Introduction

In everyday life, individuals frequently experience fluctuations in affective state, ranging from positive to negative mood. These natural fluctuations of affect can be more or less pronounced and constitute the continuous background of our actions and thoughts. Affect is defined as the simplest feeling that is consciously accessible (Russell, 2003), which varies in valence (i.e., positive versus negative) and arousal (i.e., excited versus calm). Affective states are typically prolonged states that can be caused by external triggers, such as feeling sad after watching TV news, or that occur without external causes.

Affective states have been suggested to directly influence cognitive functions such as memory or attention. Different theories have been proposed to explain the effects of negative affect on cognition. Capacity accounts, like the resource-allocation model (H. C. Ellis & Ashbrooks, 1988), proposed that negative affect is disadvantageous to cognition. According to this model, negative affect leads to the production of task-unrelated thoughts that withdraw resources from the cognitive task (Seibert & Ellis, 1991). Empirical studies have provided support for this account by reporting reduced performance on tasks measuring working memory (Figueira et al., 2017; Li et al., 2012; Spies et al., 1996), inhibition, planning (Oaksford et al., 1996; Phillips et al., 2002) and memory recall (H. C. Ellis et al., 1997; Seibert & Ellis, 1991) under negative affect compared to a neutral/positive affective state.

A different theoretical account, the affect-as-information model (Schwarz, 2011; Schwarz & Clore, 1988) suggests that people under negative affective states tend to adopt a more focused and detailed processing style, compared to a more global processing style adopted under neutral or positive affective states. This effect is thought to reduce heuristic reasoning and implicit associations, resulting in better performance in some cognitive tasks (Forgas, 2013; Forgas et al., 2005; Storbeck & Clore, 2005). Evidence for this account comes from studies finding better performance under negative affective states in false memory tasks (Storbeck & Clore, 2005, 2011) and eyewitness testimony tasks (Forgas et al., 2005), compared to positive/neutral affective states.

The contrasting theoretical views on the effects of affective states on cognition and the mixed empirical evidence in the field suggest that our understanding of the complex interplay of emotion and cognition is still very limited. To better understand the influence of negative affect on cognition, we need to use methods that can tease apart some of the underlying components of tasks. In the current study, we address the effects of emotion on prospective memory (PM), a particular memory ability involved in the realization of delayed intentions (J. Ellis & Kvavilashvili, 2000). The use of event-related potentials (West, 2011) allowed us to examine the cognitive processes underlying PM performance under different affective states in addition to behavioral measures. This approach provides further insight into both the pattern of emotion effects on cognition and potentially the underlying mechanisms.

### Prospective memory

PM is defined as the ability to perform an intended action in the future, like remembering to return a book to the library or to call a friend for his/her birthday (Kvavilashvili & Ellis, 1996). PM is crucial to maintain independence and quality of life (Woods et al., 2012) and at the same time is prone to failure (Dismukes, 2010; Niedźwieńska et al., 2020).

Given that individuals typically pursue many concurrent goals in their everyday lives, the to-be-remembered intentions are not always held in consciousness. To recreate this situation in the laboratory, participants in PM studies are invited to work on a continuous task, called the ongoing task (Einstein et al., 2005a, 2005b). An example of an ongoing task is a* 2*-back working memory task, in which participants decide on each trial whether the stimulus presented to them is the same as the one presented two trials earlier. While working on the ongoing task, participants are instructed to remember to execute a specific action when a predefined situation occurs, for example, pressing a key when a specific word appears on the screen. Researchers typically refer to these cases as event-based PM tasks in contrast to time-based tasks which are cued at a certain moment in time or after a specific duration (McDaniel & Einstein, 2007). The present study focuses on event-based prospective remembering.

PM is a process composed of multiple phases, each relying on different cognitive functions (Kliegel et al., 2002). In typical lab-based PM tasks, there is an initial encoding phase in which the intention is formed, followed by a retention phase, characterized by the maintenance of the intention and the cue that will trigger PM in long-term memory. During this phase, resources are allocated to monitor the environment for the presence of relatively low-probability PM cues. Attention must be divided across both the ongoing and the PM task. When a PM cue is detected, engagement in the ongoing task must be inhibited to switch activity to the PM intention.

### Affect-cognition interaction in PM

The resource-allocation model (H. C. Ellis & Ashbrooks, 1988) predicts that the production of task-unrelated thoughts during negative mood will be detrimental to cue detection and switching between ongoing/prospective tasks during PM paradigms. In contrast, the affect-as-information model (Schwarz, 2011; Schwarz & Clore, 1988) predicts that increased focus on task details during negative mood might enhance cue detection during PM.

The majority of studies that have investigated how negative affect influences PM have supported the predictions of the resource-allocation model. In several studies, participants performed worse on PM tasks under a negative affective state compared to a neutral affective state (Kliegel et al., 2005; Pupillo et al., 2021, 2022; Vicentin et al., 2023) and under a negative and positive affective state compared to neutral (Schnitzspahn et al., 2014). By contrast, in a study by Rummel et al. (2012), participants performed better on a PM task under negative affect compared to positive affect. The authors attributed this finding to an increased focus on the task details during negative affective states, as suggested by the affect-as-information model (Schwarz, 2011; Schwarz & Clore, 1988). Such increased focus might be beneficial for detecting a PM cue among the ongoing task. However, there was no direct evidence that negative affect enhanced cue detection.

As proposed by the resource-allocation model (H. C. Ellis & Ashbrook, 1988), one mechanism through which affective states might influence PM is the occurrence of intrusive, task-unrelated thoughts. Emotional stimuli are meaningful, can be self-relevant, and tend to be more deeply processed and therefore are more likely to elicit such thoughts (Bellana et al., 2022). These internally generated thoughts can divert limited cognitive resources away from the cognitive tasks, thereby impairing performance. Consistent with this account, Niculescu and colleagues (2024) found that trait rumination, defined as the tendency to repetitively focus on negative experiences and emotions, was associated with poor PM performance. Participants who reported ruminating more in their daily life showed greater impairments on a PM task, suggesting that stronger involvement with internal negative thoughts reduces the ability to effectively retrieve prospective intentions.

All these previous studies used behavioral data that do not allow dissociation of critical PM task components such as cue detection and task switching. We designed the present study to investigate the behavioral effects of negative affective states on PM retrieval and additionally used event-related potential (ERP) analysis, a technique that can disentangle the different cognitive processes underlying PM retrieval based on electroencephalographic (EEG) data, to examine the effects of negative affective states on the correlates of cue detection.

### Event-related potential and PM

ERPs are EEG changes that are time-locked to an event and therefore capture the time course of neural activity even in the absence of overt behavior (Kramer et al., 2006). They have been used to investigate neural activation during the different phases of PM (West, 2011; West et al., 2007). Several event-related potentials (ERPs) components have been identified as correlates of different stages of the PM process (see West, 2011, for a review), and some of them are particularly relevant to the realization of future intentions: earlier components, like the N300, the frontal positivity (FN400), and the P3b, and a late component, the prospective positivity.

The N300, a negative deflection that manifests between 200 and 500 ms after stimulus onset at the lateral occipital regions, has been related to the detection of PM cues in the environment (Cona et al., 2014; West, 2011). The lateral posterior topography of the N300 suggests that this component is generated by higher-level visual associative areas (West, 2011). Moreover, there is evidence that the N300 is reduced with increased ongoing task demands, suggesting that it might reflect the allocation of attentional resources to the detection of the PM cues (West & Bowry, 2005; West et al., 2006).

By contrast, the frontal positivity, or FN400, a positive deflection occurring between 300 and 500 ms after stimulus onset, which is coupled to the N300, seems to distinguish PM trials from ongoing task trials, and is interpreted as a familiarity-based signal that support the activation of the delayed intention once the PM cue has been detected (Cona et al., 2014; West, 2011). This component has been linked to automatic aspects of memory recollection (Cona et al., 2014; Curran, 2000).

The P3b and the prospective positivity components are instead thought to reflect functionally and temporally distinct processes that are part of a sustained positive deflection called parietal positivity (West, 2011). The P3b occurring between 300 and 400 ms after the stimulus onset reflects the detection of low-probability targets, like the PM trials, and it is associated with the evaluation of task-relevant events (Hering et al., 2016). The prospective positivity is a slow wave activity that can be seen between 400 and 1000 ms after the onset of a PM cue and is thought to be associated with the ability to switch from the ongoing to the PM task (Bisiacchi et al., 2009) and to retrieval of the PM intention (West, 2011).

### The present study

The main aim of the present study was to investigate the influence of negative affect on PM and its neural correlates, to better understand the mechanisms that might underlie any mood effects. Participants were randomly assigned to one of two affective state conditions (i.e., negative versus neutral) and worked on the same event-based PM task, while their neural activity was recorded. Using ERPs allowed us to address specific predictions of the affect-as-information and capacity accounts of negative mood effects. Specifically, if negative affect increases focus on cue detection as suggested by the affect-as-information account (Schwarz, 2011), we predict increases in the early ERP components that have been related to detection of low-probability PM cues (N300, FN400, P3b). On the contrary, if negative affect is resource-consuming as suggested by the capacity account (H. C. Ellis & Ashbrooks, 1988), we predict a reduction in the ERP correlates of cue detection and recognition (N300, FN400, P3b) and the prospective positivity component which indexes switching between ongoing and PM tasks. We investigate whether these effects of negative affect on ERP components are specific to PM trials as compared to performing ongoing task trials. Furthermore, the capacity account suggests that negative affect fosters the production of irrelevant thoughts that impair cognition (Seibert & Ellis, 1991). Thus, a secondary aim of the study was to investigate possible relationships between the amount of intrusive thoughts experienced by the participants while working on the tasks and the ERP components outlined.

## Methods

### Participants and design

Sixty-six healthy young adults volunteered in the present study.^1^ All volunteers presented normal or corrected-to-normal real vision and hearing, no history of neurological diseases, and were not suffering from current psychiatric diseases (e.g., anxiety or depression). In addition, they were not currently taking any psychopharmacological medication (e.g., antidepressants or anxiolytics). Three participants decided to withdraw from the study. Therefore, the final sample consisted of 63 young adults (*M*_*age*_ = 21.43, *SD* = 2.54, range = 17–31, 13 male, 48 female), 32 of whom were assigned to the negative mood condition (26 female, 6 male), and 31 to the neutral mood condition (24 female, 7 male). The two groups did not differ in relation to self-reported health (measured on a 5-point Likert scale; *M* = 3.89*, SD* = 0.75), crystallized intelligence (*M* = 15.34*, SD* = 3.70) measured with the Mill-Hill Vocabulary Scale (Raven, 1983), and fluid intelligence (*M* = 64.64*, SD* = 10.41) measured via the Digit-Symbol Substitution Test (Wechsler, 2008), *p*s >.182. Handedness was measured with the Edinburgh Handedness Inventory (Oldfield, 1971). Results of the Handedness Inventory showed that 55 participants were right-handed (28 assigned to the neutral affect condition, 27 to the negative affect condition), five ambidextrous (three assigned to the neutral affect condition, two to the negative affect condition), and three left-handed (one assigned to the neutral affect condition, two to the negative affect condition). Most participants were undergraduate students at the University of Aberdeen, who received course credits in exchange for participation. The remaining participants were volunteers who were reimbursed £20 for their participation.

### Procedure and materials

Volunteers were tested individually in one session which lasted about two hours. First, participants’ sociodemographic information (age, gender, general health, education, and handedness) was gathered. Participants were asked to perform the Mill-Hill Vocabulary Scale and the Digit-Symbol Substitution Test and then were fitted with the electrodes for the EEG measurement. The application of the cap and the electrodes took approximately 30 min. Participants were then given instructions for the computerized tasks, and the first assessment of mood took place. Participants’ mood was assessed several times throughout the experiment: before starting the cognitive computerized tasks, after each film clip, and at the end of the computerized tasks.

The basic procedure for the study was as follows (with further details of each task in the sections below). After the first mood measure, in which the Self-Assessment Manikin Scale (SAM, Bradley & Lang, 1994) was filled in, participants were given instructions for the ongoing working memory 2-back task and were invited to perform the practice block of 12 ongoing trials. The practice trials were followed by an ongoing-task-only block. After completing this block, participants were given instructions for the PM task, filled in the second SAM and were invited to watch the first film clip which served as affective state manipulation.

Participants watched either a negative or a neutral film clip, depending on the condition they were assigned to. At the end of the first film clip, they rated their mood again and started working on the first PM block. This sequence of film clip, SAM, and PM block was repeated three more times until participants watched all four film clips and completed all four PM blocks. Participants watched four film clips of the same valence (negative or neutral) throughout the study. The order of the blocks was randomized and was different for each participant.

After performing the PM tasks, participants completed the last SAM and recalled the task instructions and the PM cues. Subsequently, they completed a short post-test questionnaire.

#### Affective state assessment

To assess participants’ affective state, we used computerized versions of the valence and arousal subscales of the SAM. The SAM is a nonverbal questionnaire, which includes figures depicting emotions, ranging from happy to unhappy for the valence subscale, and from excited to calm for the arousal subscale. This measure was chosen for several reasons. First, the SAM measures dimensional affective state, rather than categorical emotions, reflecting our interest in inducing and measuring affective states which vary on the neutral-negative dimension. Second, the SAM is a brief measure of affective *state*, as opposed to affective *trait*, widely used in various settings and populations, and thus it is considered to be a reliable assessment of transient emotions (Bynion & Feldner, 2017). Finally, the valence subscale of the SAM has been previously used in affect-PM studies to reliably measure changes in affective states following mood induction procedures (e.g., Pupillo et al., 2021; Rummel et al., 2012). In the present study, the SAM was displayed on the computer screen. Scoring ranged from 1 (very unhappy) to 100 (very happy) for the valence subscale, and 1 (very calm) to 100 (very excited) for the arousal subscale. Filling in the SAM was required to continue with the experiment.

#### Ongoing tasks

Similarly to previous mood-PM studies (Pupillo et al., 2021, 2022; Schnitzspahn et al., 2014), the PM task was embedded in a 2-back working memory task that served as the ongoing task. 81 different pictures were taken from the Snodgrass and Vanderwart (1980) standardized set and assigned either to the PM task or the ongoing task. The images depicted emotionally neutral objects as black outlines against a white background.

**Fig. 1:**
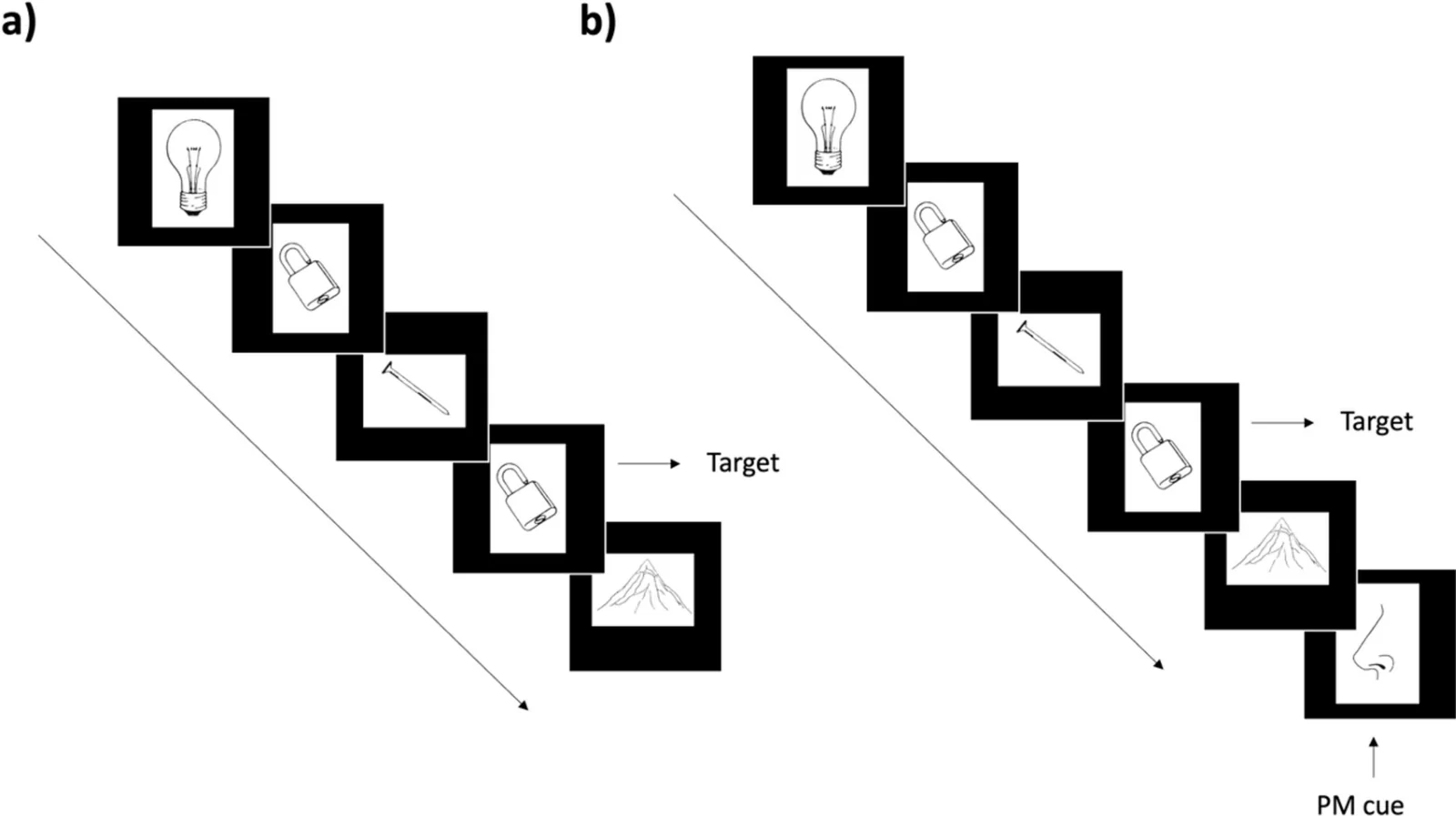
Ongoing (2-back) and prospective memory (PM) tasks. *Note*. Depiction of (**a**) the ongoing (2-back) and (**b**) the PM task. In the ongoing task, participants had to press the “yes” button on the keyboard every time the picture presented was the same as the one that had appeared two trials before (Target). If the picture did not match the one presented two trials before, they had to press the “no” button on the keyboard. Each picture was presented for 1000 ms and preceded by a fixation cross of 300 ms. In the PM task, the PM cue was embedded in the 2-back task. Participants had to additionally remember to press the “space” bar every time one of six pictures that were previously encoded appeared on the screen (PM cue)

In the ongoing 2-back task, pictures were presented consecutively on the computer screen (see Fig. 1). At the start of the task participants were asked to place the right middle finger on the “no” button and the right index finger on the “yes” button. Participants were instructed to press the “yes” button on the keyboard every time the picture presented was the same as the one that had appeared two trials before. If the picture did not match the one presented two trials before, they had to press the “no” button on the keyboard. Each picture of the ongoing task was presented for 1000 ms and preceded by a fixation cross of 300 ms. After each picture, a black screen of 1200 ms was presented. Participants could select a response during the 2200 ms that followed the stimulus onset.

Each of the 81 different pictures was repeated seven times throughout the experiment. The order of the pictures was randomized, with the restriction that in one-third of the trials the picture matched that presented two trials previously (ongoing 2-back task target trials) and in two-thirds they did not match the picture presented two trials before (ongoing task non-target trials). The 2-back task was presented in six blocks: the practice block, the ongoing task-only block and four PM blocks. The practice block was composed of 12 trials, the ongoing task-only block of 54 trials, and the PM blocks were composed of 101 trials each.

In the practice trials, every response was followed by on- screen feedback informing participants whether the response given was correct or incorrect, while in the ongoing-task-only feedback was not provided. To proceed to the next stage of the experiment, participants had to reach an overall performance of 80% correct in the practice trials.

#### Prospective memory task

For the PM task, we used an event-based task with some images from the ongoing task as cues. After completing the initial ongoing-task-only block, participants were presented with the six images which acted as the PM cues. The cue images represented a mouse, a hat, a nose, an orange, a book, and a wineglass, respectively. These particular images were chosen because they all belonged to different semantic categories (i.e., animal, clothing, household objects, body parts, fruit, stationery), all of which were represented among the ongoing task stimuli. Therefore, we assumed that the PM images were not more salient nor more distinct than the ongoing task images.

Participants were instructed to memorize the PM images, which were presented twice, each time for 1000 ms, with a blank screen of 1500 ms between the pictures. To ensure successful encoding, after the presentation of the images, participants were invited to recognize the cues among distractors in a brief recognition task. Once all the PM cues were correctly recognized, participants proceeded to the next experimental task. Participants were then instructed to press the “space bar” every time the picture presented on the screen was one of those six images they were asked to memorize (the “cues” triggering prospective remembering). They were asked to press the “space bar” for the PM cues instead of the “yes” and “no” buttons. The experimenter invited participants to repeat the instructions in their own words, to ensure correct understanding of the task.

Note that the event-based PM paradigm bears some resemblance to an oddball paradigm, as it is based on the detection of infrequent events. However, whereas an oddball paradigm is designed to capture the detection of stimuli which are perceptually different from frequent ones (Squires et al., 1975), in event-based PM paradigms, the cues are not different from the rest of the stimuli and task performance relies on the ability to recognize the retrieval cue, retrieve the action which was linked to the intention, and perform the action (J. Ellis & Kvavilashvili, 2000).

Each PM cue was repeated seven times across the four PM blocks, resulting in 42 PM trials in total (6 cues × 7 repetitions). Two PM blocks contained 11 PM cues each, while the other two blocks contained 10 PM cues each. In between each of the four PM blocks the film clips used to manipulate mood were presented. The order of the blocks was randomized and was different for each participant.

#### Affective state manipulation

Affective state was manipulated by presenting participants with film clips of neutral and negative valence, respectively. We chose this mood induction methods because it was utilized successfully in previous affect-PM studies (Kliegel et al., 2005; Pupillo et al., 2021; Rummel et al., 2012; Schnitzspahn et al., 2014) and because it is considered effective, especially for inducing negative affective states (Fernández-Aguilar et al., 2018). The film clips were taken from movies and documentaries and lasted about three minutes. Each film clip was edited to create a coherent story and has been used in a previous study to successfully induce negative and neutral affective states in young adults (Pupillo et al., 2021). Examples of film clips used for the negative affective state induction are a hanging scene from the movie “Dancer in the Dark” and an electric chair execution from “The Green Mile”. Examples of clips used for the neutral affective state condition are an extract from a documentary about camels and images of a tropical beach. Additional information on the film clips, including valence and arousal mean ratings obtained in a pilot study, can be found in Supplementary Materials (Table S1).

Previous studies showed that the induced negative affective state tends to slightly decrease after around 10 min (Kliegel et al., 2005). The relatively short duration of our ongoing task blocks, which lasted around 8 min, should mean that the induced affective state was maintained for the duration of the task blocks.

Following previous studies (Rummel et al., 2012; Schnitzspahn et al., 2014) we identified mood non-responders depending on participants’ reported affective states. The average valence score for participants in the neutral condition after watching the film clips was used as a cut-off score. Participants who scored higher on average than the cut-off after watching the negative film clips (*M* = 67.50, *SD* = 11.56) were excluded from further analyses. One participant in the negative condition who reported mean valence higher than the cut-off score after watching the negative film clips (*M* = 90.75, *SD* = 0.95) was excluded from further analyses.

#### Post-test questionnaire

After the task, participants were asked again to recognize the PM cues among distractors. Then, in a short post-test questionnaire, they were asked to indicate whether they used any strategy to perform the memory tasks or to reduce their emotional reactions toward the content of the film clips, and to recall the six PM cues. In addition, they were also asked to rate to what extent they experienced intrusive thoughts related to the film clips while doing the cognitive tasks, on a scale from 1 (not at all) to 7 (a great deal).

### Recording and analysis of electrophysiological data

EEG data were recorded continuously during the ongoing task-only block and the four PM blocks using the BioSemi ActiveTwo system (Biosemi, Netherlands) with 64 Ag/AgCl active scalp electrodes. Electrodes were positioned according to the 10–20 international system. In addition, four facial electrodes were applied near the outer canthi on the left and right side and below and above the left eye, to monitor eye movements. The BioSemi ActiView software was used to record data (BioSemi, V.O.F., Amsterdam, Netherlands), which were digitized at a sampling rate of 2048 Hz in a bandwidth filter of 0–417 Hz. Processing of the data was carried out offline via a fully automated script using the EEGlab 14.1.2 software (Delorme & Makeig, 2004) for Matlab (Version R2017a, MathWorks, Natick, MA, United States). Post-processing, EEG was visually inspected in EEGLAB to confirm that artifacts were correctly removed and that no residual high-amplitude noise remained.

The analysis pipeline is documented in a Matlab script which includes all parameter settings used for preprocessing (see Supplementary Materials). First, data were downsampled to 256 Hz prior to analysis and then segmented in relation to the position of the stimulus onset markers in time windows of −200 to 1300 ms. Only trials with a correct response were selected. Data were lowpass filtered at 40 Hz using a Finite Impulse Response (FIR filter) with a Kaiser window. To avoid potential latency and low-frequency distortions, we did not apply high-pass filtering. To correct for artifacts, the FASTER algorithm was used (Nolan et al., 2010). FASTER is an automated procedure that calculates statistical parameters of the data to detect noisy channels and globally contaminated trials that need to be rejected or locally contaminated trials that need interpolation. The FASTER procedure characterizes channel properties (amplitude range, deviation from channel average, etc.) using statistical parameters of the data and applies a metric of ± 3 *z*-score as a marker for contaminated data. First, we rejected globally contaminated trials, namely the trials with excessive noise that need to be removed prior to Independent Component Analysis (ICA). On average, 9.51 globally contaminated epochs per participant were rejected by the FASTER algorithm, corresponding to around 2% of trials per participant (*SD* = 3.54). ICA was then performed with the RunICA, extended—Infomax algorithm, and the ADJUST toolbox (Mognon et al., 2010) was used to identify components driven by blinks, eye movements and local discontinuities by evaluating the properties of obtained maps against artifact-specific spatial and temporal features. These artifactual ICA components were subtracted from the data. While ICA decomposition benefits from application of a high-pass filter, we avoided high-pass filtering due to its effect on ERP component latencies (e.g., van Driel et al., 2021). Instead, we ensured adequate data quality during the recording, with a comfortable room temperature that precluded low-frequency drifts due to sweating, and with a relatively short duration of the recording, which was between 30 and 35 min. As a further step, we also rejected any trials with blinks or eye movements as identified by application of amplitude range criteria to VEOGs and HEOGs computed from detrended eye channel data (80 microvolt for blinks, 25 microvolts for eye movements). On average, around 2 blink components (*SD* = 0.9), 2 eye movement components, and 3 discontinuity components (*SD* = 1.9) were removed per participant. An average of 1 blink-contaminated trial (*SD* = 3.31) and 25 eye movement-contaminated trials (*SD* = 26.8) were excluded. Overall, an average of 36 trials were removed per participant (*SD* = 28.13), corresponding to 9% of the total trials. Finally, we used FASTER to identify and interpolate globally (i.e., throughout the recording) and locally (i.e., single epoch-specific) contaminated channels. Before ERP analyses, all electrodes were re-referenced to the average reference. Baseline correction was applied using 100 ms pre-stimulus activity.

ERP averages were computed for trials with correct responses (i.e., hits) elicited by the three events of interest within the PM blocks: ongoing task targets, ongoing task non-targets, and PM targets. Including only correct trials allowed us to isolate neural activity associated with successful task performance, avoiding contamination from error-related processes. Furthermore, restricting analyses to PM blocks ensured that all trials were embedded within a consistent dual-task context, thereby minimizing confounds related to differences between single-task and dual-task conditions. ERPs were averaged for each subject and condition and quantified as measures of mean amplitude of the component of interest. Time windows and electrode sites for the mean amplitudes were selected according to the existing literature (Cona et al., 2014; Hering et al., 2018; West, 2011) and visual inspection of the grand averages. For the early components (N300, FN400, P3b), time windows for mean amplitude calculation were centered at peak latencies of the grand-mean ERPs. For prospective positivity, mean amplitude within the time window was considered. We analyzed the N300 within 220–420 ms averaged across electrodes O1, OZ and O2. The P3b was analyzed within 230–400 ms averaged across the electrode cluster containing POz, P1, CPz, P2 and Pz. The FN400 was analyzed within 300–600 ms at electrode cluster containing FCz, F1, Afz, F2 and Fz. The prospective positivity was analyzed within 450–900 ms at electrode cluster containing Pz, CP1, Cz, CP2 and CpZ.

### Statistical analysis

Statistical analyses were performed in R (version 4.4.2; R Core Team, 2025) with the *lme4* package (Bates et al., 2014).

To check if the mood manipulation was effective, the valence and arousal scores were analyzed with linear mixed-effects models, with affective state condition (negative versus neutral, between-participants), timepoint (first, second, third; within-participants), and their interaction as fixed effects, and participant as random intercept. The mean of the first two affect measures was used as baseline (first timepoint), while the mean of the four mood measurements after the film clips was used as a measure of mood after the induction (second timepoint). Finally, the last affective state measurement after completion of the PM task was also included (third timepoint).

Before starting the behavioral analysis, we verified that all participants correctly remembered the PM task instructions at the end of the testing section. One participant assigned to the neutral mood condition who did not follow the instructions and did not press any key in the ongoing task of the PM blocks was excluded from further analyses. In addition, we explored the role of intrusive thoughts by regressing PM performance on the level of intrusive thoughts experienced by participants while working on the tasks across both mood conditions.

Behavioral data were analyzed using logistic mixed-effects models to appropriately account for the binary outcome structure. Behavioral analyses of ongoing task performance included trials from both the ongoing task-only block and the PM blocks, with block type modeled as a within-participant factor and included as a random slope, while affective state condition was included as a between-participant factor. In contrast, ERP analyses were restricted to trials within the PM blocks.

Averaged amplitudes for P3b, prospective positivity, and FN400 were analyzed using linear mixed-effects models. Each model included affective state (negative versus neutral; between-participants), trial type (ongoing task target trials, ongoing task non-target trials, PM trials; within-participants), and their interaction as fixed effects, with random intercepts for participants. Trial type was included to compare ongoing task and PM modulations and confirm PM-specific ERP modulations. Significant interactions were followed by post-hoc *t*-tests. All *p*-values were multiplied by the number of comparisons made (indicated as *p*_*adj*_). Sensitivity analyses for the main effects observed are reported in the Supplementary Material.

All linear mixed-effect models included participants as random intercepts to capture baseline differences. *F*-tests, likelihood-ratio χ^2^ (for logistic regression), degrees of freedom, and *p*-values were obtained via Satterthwaite approximation. Post‑hoc contrasts were conducted using the *emmeans* package (Lenth, 2017).

In addition, we computed exploratory bootstrapped correlations between intrusive thoughts and ERP components. Given the exploratory nature of the analysis, we report uncorrected *p*-values.

For all tests, Bayes factors (*BF*_*10*_) were computed using the Savage–Dickey density ratio using the *brms* package (Bürkner, 2017) to quantify evidence for a nonzero effect relative to a null hypothesis. In addition, for theoretically motivated directional hypotheses (i.e., contrasting predictions of the resource-allocation model and the mood-as-information model), posterior probabilities and evidence ratios (ERs) are reported. Evidence ratios quantify the relative posterior support for one direction of an effect over the opposite direction (e.g., *P*(*β* < 0 | *data*)/*P*(*β* > 0 | *data*)), indicating how much more strongly the posterior distribution supports a negative relative to a positive effect. (Wagenmakers et al., 2010). Unlike conventional Bayes factors, which typically compare a null hypothesis against an alternative hypothesis, ERs evaluate the relative support for competing directional predictions. ERs were also used to estimate directional hypotheses for planned contrasts (e.g., *P*(*δ* < 0 | *data*)/*P*(*δ* > 0 | *data*)). Bayes factors were interpreted using conventional guidelines in psychological research (Wagenmakers et al., 2018): values of 1–3 indicate anecdotal evidence, 3–10 moderate evidence, 10–30 strong evidence, 30–100 very strong evidence, and values greater than 100 extreme evidence for the alternative hypothesis, with reciprocal values indicating evidence for the null. ERs were interpreted analogously (e.g., ER = 10 indicates that one direction is ten times more supported than the other). Because the priors were symmetric around zero, the reported ERs are numerically equivalent to Bayes factors for the corresponding directional hypothesis or parameter comparisons (Kass & Raftery, 1995).

## Results

### Affective state manipulation

One participant in the negative condition who scored higher than the cut-off score after watching the negative film clips was excluded from further analyses. Of the 61 remaining participants: 31 were assigned to the negative and 30 to the neutral affective state condition.

**Fig. 2:**
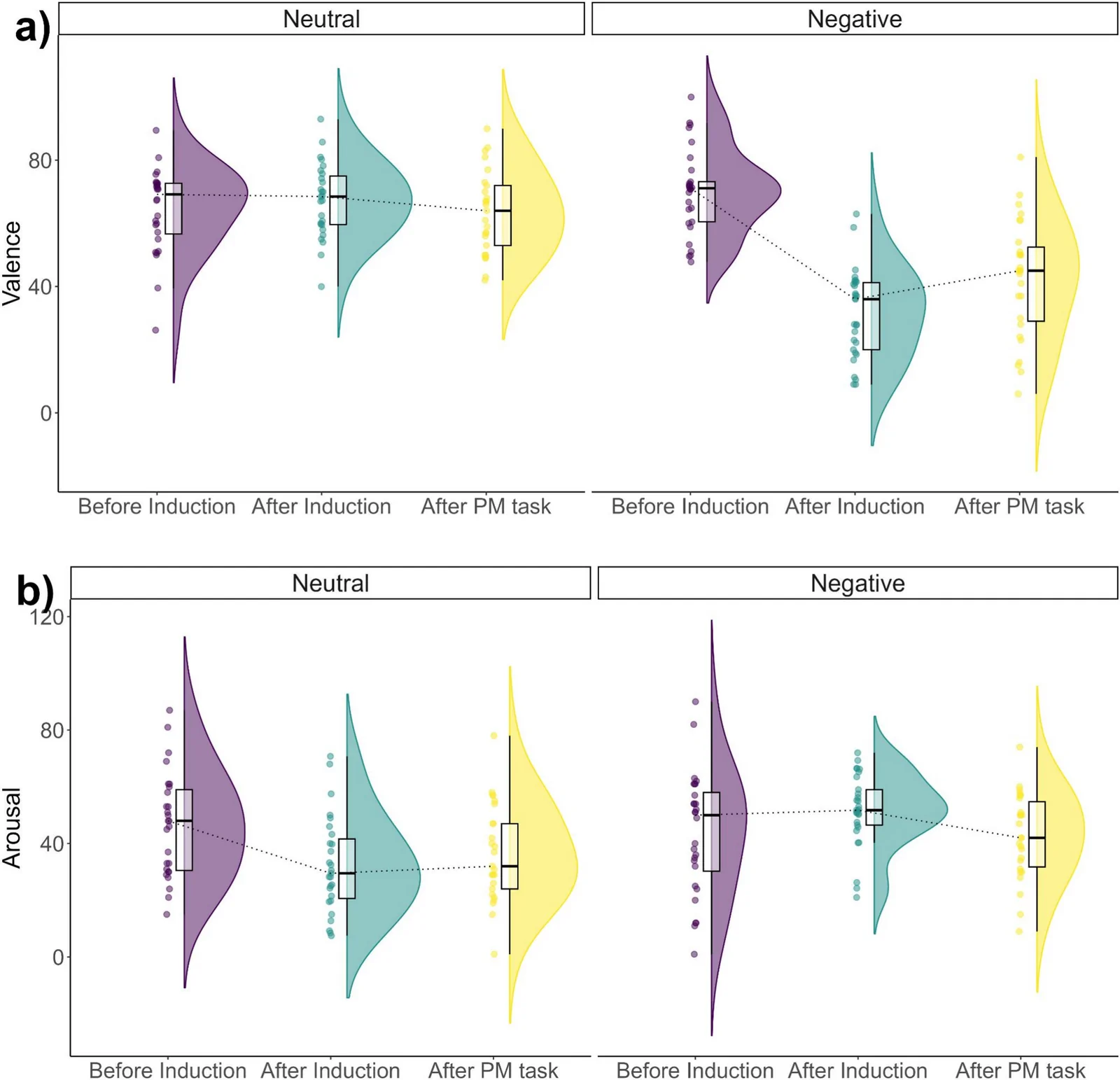
(**a**) Valence and arousal as a function of affective state condition and timepoint. *Note*. These plots represent the aggregated valence and arousal scores of the SAM as a function of affective state condition and timepoint, for (**a**) valence and (**b**) arousal. The valence subscale ranged from 1 (very unhappy) to 100 (very happy), while the arousal subscale ranged from 1 (very calm) to 100 (very excited)

Valence and arousal scores as a function of affective state condition and timepoint are presented in Fig. 2. For valence, there were strong main effects of affective state condition, *F*(1,60.79) = 36.25, *p* <.001, ${\eta}_{p}^{2}$ =.38, *BF*₁₀ > 100, and timepoint, *F*(2,117.36) = 56.76, *p* <.001, ${\eta}_{p}^{2}$ =.49, *BF*₁₀ > 100, as well as a significant interaction, *F*(2, 117.36) = 61.33, *p* <.001, ${\eta}_{p}^{2}$ =.51, *BF*₁₀ > 100.

Follow-up analyses showed that participants’ mood before the affective state induction did not differ across neutral and negative conditions, *t*(60) = 1.24, *p*_*adj*_ = 0.629, *d* = 0.31, *BF*₁₀ = 0.26, indicating evidence for the absence of a difference. After the induction, participants in the negative condition (*M* = 32.14, *SD* = 14.04) reported substantially lower valence than participants in the neutral condition, (*M* = 67.37 *SD* = 11.35), *t*(59) =− 9.95, *p*_*adj*_ <.001, *d* =—2.79, *BF*₁₀ > 100, providing extreme evidence for an effect. The effect of the affective state induction remained even after the PM task was finished, *t*(58) =− 6.05, *p*_*adj*_ <.001,* d* = − 1.39, *BF*₁₀ > 100.

For arousal, there was a main effect of timepoint, *F*(2,59.84) = 4.09, *p* =.048, ${\eta}_{p}^{2}$ =.05: however, the corresponding Bayes factor indicated anecdotal evidence against including timepoint (*BF*₁₀ = 0.40), suggesting that this effect is weak. Similarly, there was a main effect of affective state condition, *F*(2,59.96) = 4.05, *p* =.037, = ${\eta}_{p}^{2}$.06, but the Bayes factor indicated only anecdotal evidence *BF*₁₀ = 1.29. In contrasts, there was strong evidence for an affective state condition by timepoint interaction, *F*(2,114.87) = 10.67, *p* <.001, ${\eta}_{p}^{2}$ =.16, *BF*₁₀ > 100. Planned contrasts revealed that before the mood induction arousal did not differ across conditions, *t*(60) = −1.033, *p*_*adj*_ =.910,* d* = −0.21, *BF*₁₀ = 0.29, indicating evidence for the absence of a difference. After the induction, arousal was higher in the negative condition (*M* = 51.66, *SD* = 12.34) than in the neutral condition (*M* = 33.37, *SD* = 16.43), *t*(60) = 3.99, *p*_*adj*_ <.001,* d* = 1.16, *BF*₁₀ > 100, indicating strong evidence for an effect*.* After the PM task, the difference was no longer supported, *t*(60) = 1.68, *p*_*adj*_ =.284,* d* = 0.48, *BF*₁₀ = 0.57.

To summarize, those in the negative mood condition reported more negative valence and higher arousal than those in the neutral condition immediately after the mood induction. The difference in valence was maintained to the end of the cognitive tasks, whereas arousal differences were transient.

### Behavioral results

#### PM task

**Fig. 3:**
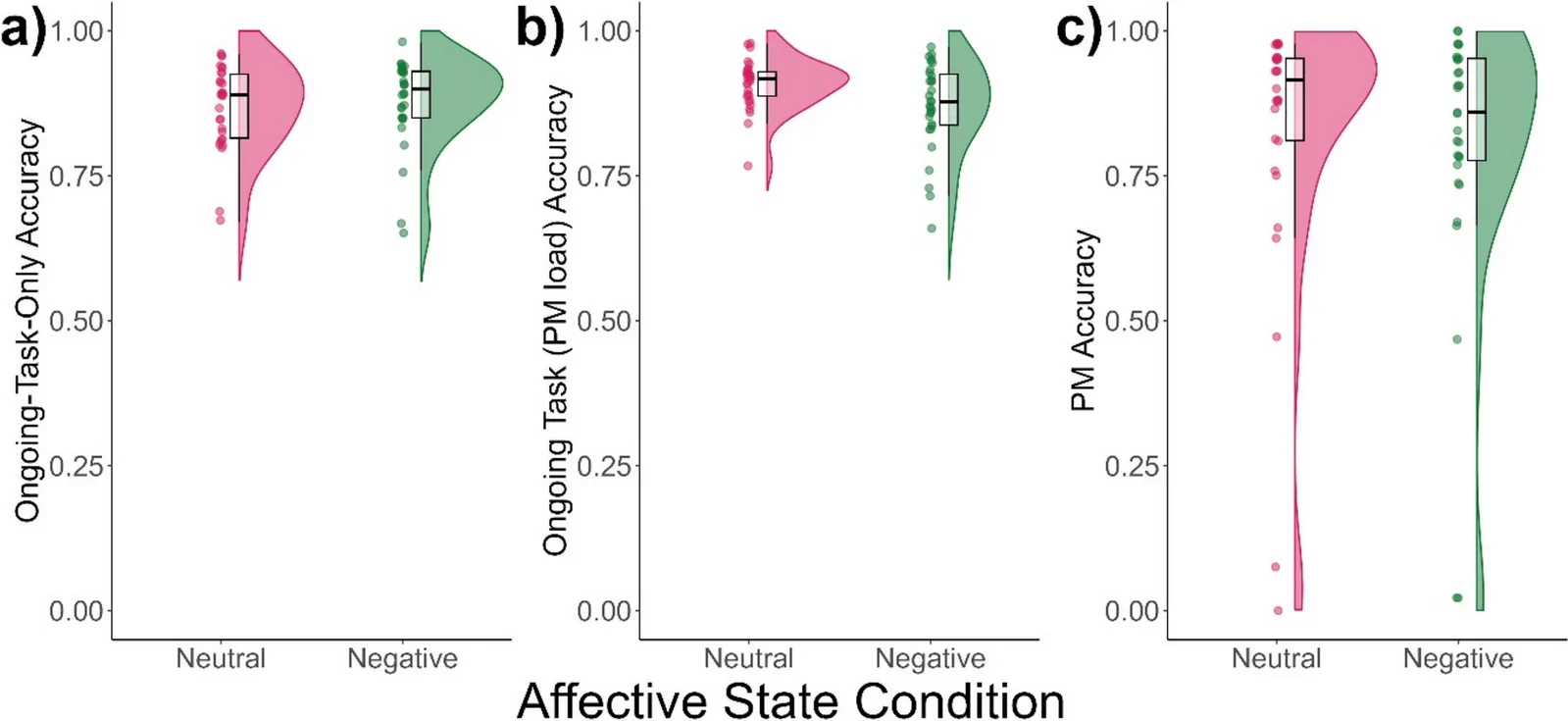
Ongoing and PM task accuracy and affective state condition. *Note.* The figure represents (**a**) the ongoing-task-only block, (**b**) the ongoing task with the PM load, and (**c**) PM accuracy as a function of affective state condition

PM accuracy of participants assigned to the negative affective state condition (*M* = 0.82, *SD* = 0.24) did not differ from accuracy of participants assigned to the neutral affective state condition (*M* = 0.80, *SD* = 0.24), *χ*^*2*^(1) = 0.24, *p* =.625, *BF*₁₀ = 0.73, indicating no clear support for a difference (see Fig. 3). The posterior distribution provided only anecdotal support for a decrease in PM in the negative vs neutral condition *P*(*β* < 0 | *data*) = 0.63 (ER = 1.68). As the plots showed that four participants presented PM accuracy close to zero, the analysis was rerun after excluding these outliers (two participants in the negative and two in the neutral condition). PM accuracy of participants assigned to the negative affective state condition (*M* = 0.88, *SD* = 0.12) still did not differ from accuracy of participants in the neutral affective state condition (*M* = 0.86, *SD* = 0.12), *χ*^*2*^(1) = 0.76, *p* =.383, *BF*₁₀ = 0.68). The posterior distribution provided moderate directional support for reduced PM performance in the negative vs neutral condition, *P*(*β* < 0 | *data*) = 0.76, corresponding to an ER of 3.15 (see Fig. 7a). There were also no group differences for the number of previous PM cues correctly remembered in the final recall (negative condition: *Mdn* = 5, range = 2–6; neutral condition: *Mdn* = 5, range = 3–6), *t*(60) = −0.63, *p* =.533, *d* = − 0.16, *BF*₁₀ = 0.31, and the final recognition task (negative condition: *M* = 0.94, *SD* = 0.12; neutral condition:* M* = 0.97, *SD* = 0.06), *t*(60) = 1.27, *p* =.210, *d* = − 0.33, *BF*₁₀ = 0.06, both providing evidence for the absence of meaningful differences.

#### Ongoing task

There was no main effect of affective state condition on the ongoing task, *χ*^*2*^(1) = 1.39, *p* =.239, *BF*₁₀ = 0.57. There was a main effect of block type, *χ*^*2*^(1) = 6.16, *p* =.013, *BF*₁₀ = 2.04, indicating anecdotal evidence for slightly better performance in PM blocks (*M* = 0.89, *SD* = 0.06) compared to the ongoing task-only block (*M* = 0.87, *SD* = 0.07). There was a significant block by affective state interaction, *χ*^*2*^(1) = 7.10, *p* =.008, *BF*₁₀ = 5.97, indicating moderate evidence that the effect of condition differed across blocks. To follow up on the interaction, two independent *t-*tests were carried out separately for the two blocks (ongoing task-only and PM blocks). In the ongoing task-only block, there was no difference between the two affective state conditions, *z* = 0.21, *p*_*adj*_ =.833, *d* = 0.04, *BF*₁₀ = 0.33, while in the PM blocks participants in the neutral condition (*M* = 0.91, *SD* = 0.04) performed better than participants in the negative condition (*M* = 0.87, *SD* = 0.07), *z* = 2.54, *p*_*adj*_ =.011, *d* = 0.63, *BF*₁₀ = 4.17, indicating moderate evidence for a difference.

To summarize the behavioral results across PM and ongoing task blocks, there was no effect of affective state on PM performance, but those in the negative affective state condition performed worse than those in the neutral condition on the ongoing task blocks with the additional PM load.

### Electrophysiological data

**Fig. 4:**
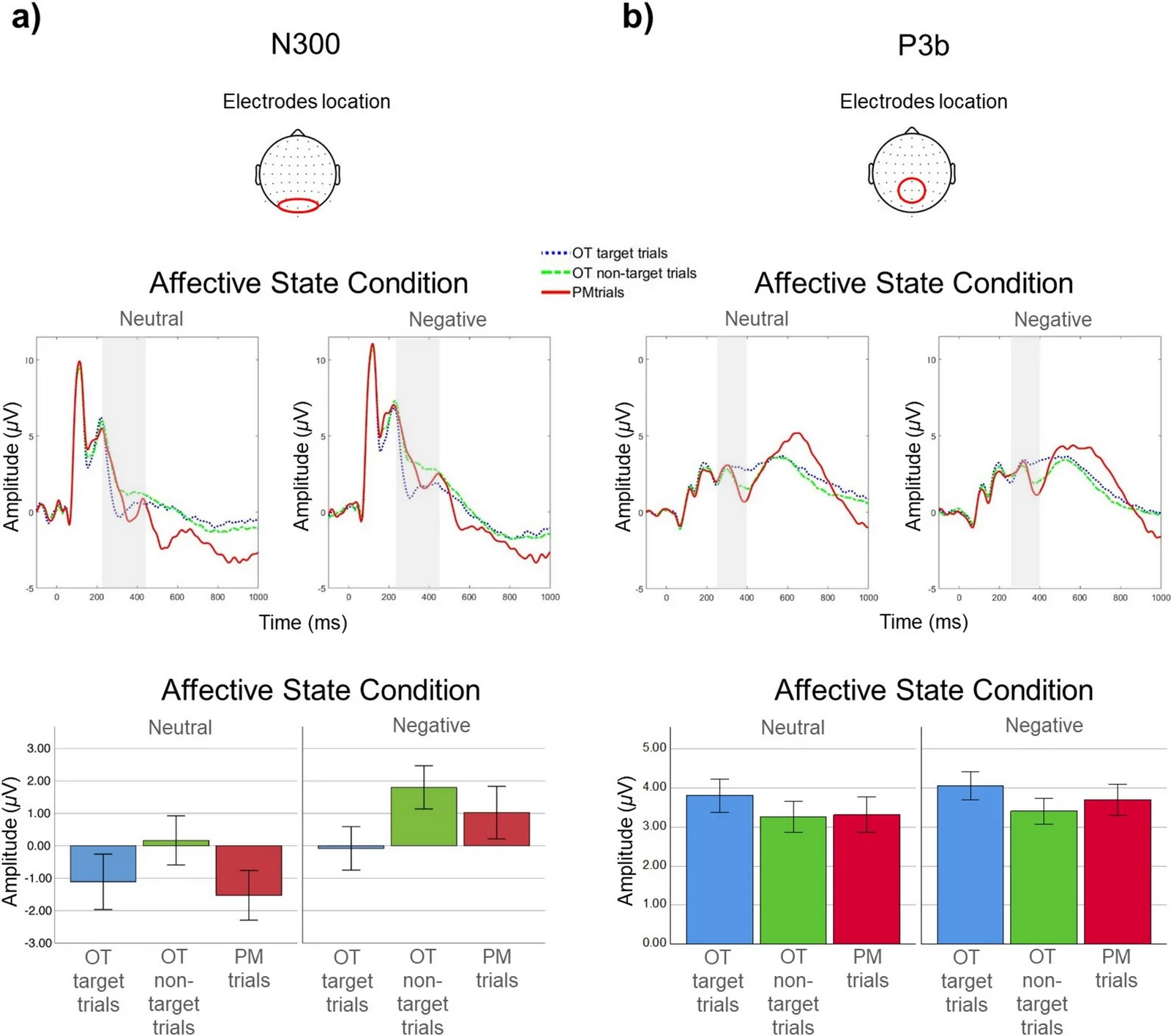
ERP grand means of N300 and P3b amplitudes. *Note.* Location of the selected electrodes for each component and ERP waveforms from the electrodes of interest are shown. The different colors and shapes of the waveforms represent the trial type: The blue dotted line represents the ongoing task (OT) target trials; the green dashed line represents the OT non-target trials; the red solid line represents the PM trials. The shaded areas show the selected time window. On the bottom part, bar graphs show the mean amplitude as a function of trial type and affective state condition

**Fig. 5:**
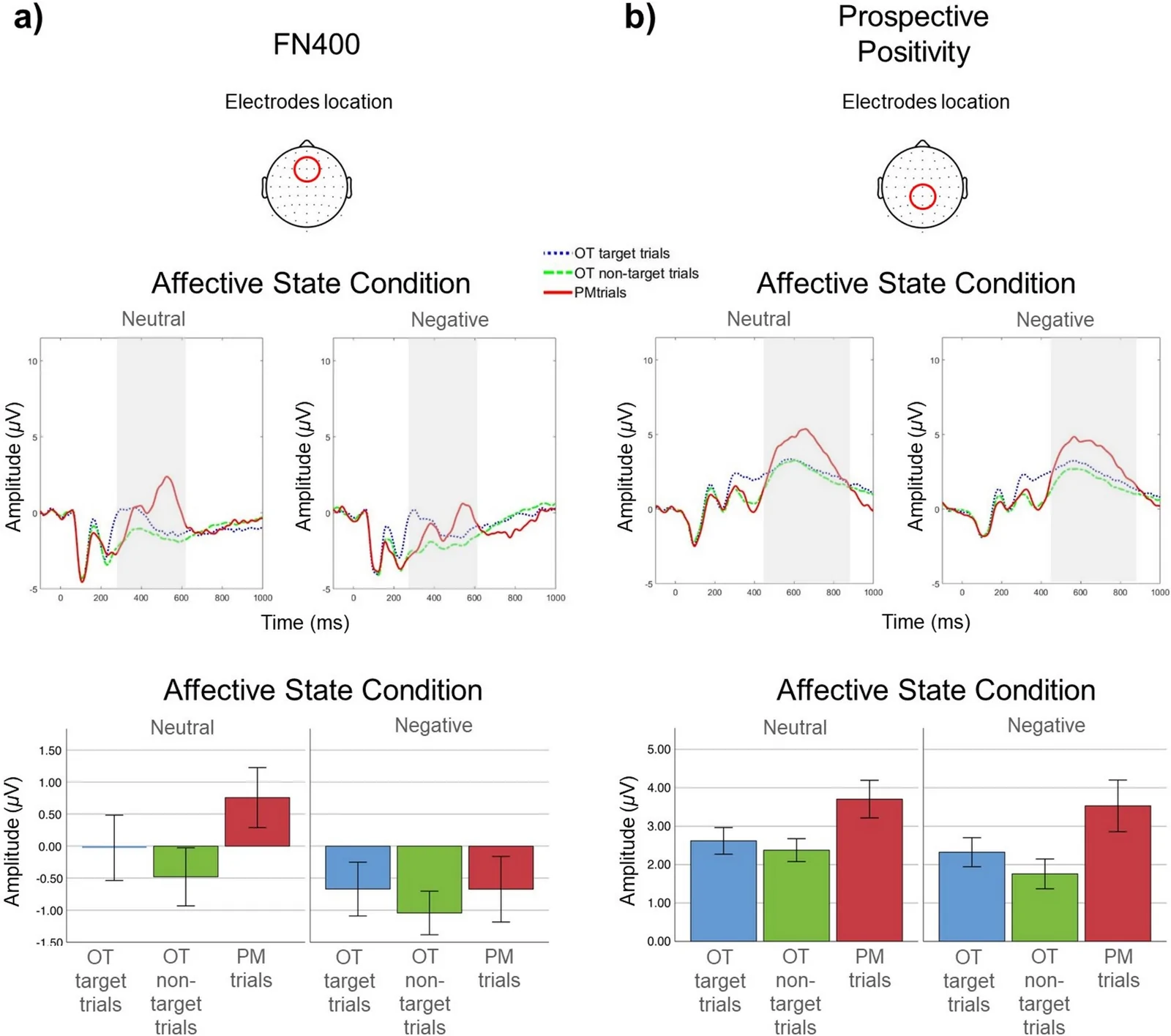
ERP grand means of FN400 and prospective positivity amplitudes. *Note*. Location of the selected electrodes for each component and ERP waveforms from the electrodes of interest are shown. The different colors and shapes of the waveforms represent the trial type: The blue dotted line represents the ongoing task (OT) target trials; the green dashed line represents the OT non-target trials; the red solid line represents the PM trials. The shaded areas show the selected time window. On the bottom part, bar graphs show the mean amplitude as a function of trial type and affective state condition

The grand means for the N300 and P3b, as well as FN400 and Prospective Positivity, as a function of trial type and mood condition, are shown in Figs. 4 and 5, respectively. Descriptive data for the components of interest and inferential results for the components of interest are reported in Table S2 and Table S3 in the Supplementary Material.

#### N300 amplitude

For the N300 amplitude, results showed a strong main effect of trial type, *F*(2,116) = 20.36, *p* <.001, ${\eta}_{p}^{2}$ =.26, *BF*₁₀ > 100. The interaction between affective state condition and trial type also reached significance, *F*(2,116) = 4.18, *p* =.018, ${\eta}_{p}^{2}$ =.07, *BF*₁₀ = 3.57, indicating moderate evidence (see Fig. 4a for grand-average waveform). The main effect of affective state condition was not supported, *p* =.180, *BF*₁₀ = 1.18. Three separate *t*-tests were carried out for the amplitudes of three different trial types (ongoing task target trials, ongoing task non-target trials, and PM trials). Results showed that the N300 amplitude of the ongoing task target trials and ongoing non-target trials did not differ between the negative and the neutral condition (ongoing task target trials: *t*(58) = 0.61, *p*_*adj*_ = 1.00, *d* = 0.15; *BF*₁₀ = 0.54; ongoing task non-target trials: *t*(58) = 1.15, *p*_*adj*_ =.758 *d* = 0.32, *BF*₁₀ = 0.69). For the PM trials, the contrast did not survive correction, *t*(58) = 2.28, *p*_*adj*_ =.114, *d* = 0.52, and the Bayes factor indicated anecdotal evidence for a nonzero effect (*BF*₁₀ = 2.78). However, the posterior distribution of this contrast was strongly concentrated below zero, *P*(*δ* < 0 | *data*) = 0.97 (ER = 32.71; see Fig. 7b). This pattern suggests that, while the evidence for an effect was limited, as indicated by the BF and frequentist analysis, the posterior distribution indicated substantially greater support for a larger (more negative) N300 response in the neutral relative to the negative mood condition.

#### P3b amplitude

For the P3b amplitude, the analysis revealed a strong main effect of trial type, *F*(2,116) = 7.67, *p* <.001, ${\eta}_{p}^{2}$ =.12, *BF*₁₀ = 41.60, but no effect of affective state condition or interaction, (*p*s >.61, *BF*_*S*_₁₀ < 1; see Fig. 4b). Pairwise analyses showed evidence against the difference in amplitude between PM trials and ongoing task non-target trials, *t*(116) = 0.19, *p*_*adj*_ = 1.00, *d* = 0.02, *BF*₁₀ = 0.17. In contrast, there was strong evidence for ongoing task target trials being higher than both the ongoing task non-target trials, *t*(116) = 3.31, *p*_*adj*_ =.003, *d* = 0.32, *BF*₁₀ = 25, and PM trials, *t*(116) = 3.49, *p*_*adj*_ =.002, *d* = 0.31, *BF*₁₀ = 50.

#### FN400 amplitude

The linear mixed-effect model for the FN400 showed a strong main effect of trial type, *F*(2,116) = 49.27, *p* <.001, ${\eta}_{p}^{2}$ =.46, *BF*₁₀ > 100, and a trial type x affective state interaction, *F*(2,116) = 3.47, *p* =.034, ${\eta}_{p}^{2}$ =.06, *BF*₁₀ = 2.24, indicating anecdotal evidence. The main effect of affective state condition was not supported, *F*(2,116) = 2.51, *p* =.333, ${\eta}_{p}^{2}$ =.02, *BF*₁₀ = 0.76. To break down the interaction, we compared negative vs. neutral within each trial type. For ongoing task target and ongoing task non-target trials, there were no mood-related differences (both *p*_*adj*_ = 1.000, BFs₁₀ < 1). For PM trials, the FN400 amplitude in the negative condition (*M* = − 1.06, *SD* = 2.51) was smaller than in the neutral condition (*M* = 0.56, *SD* = 2.55). The contrast did not survive correction, *t*(55) = − 1.46, *p*_*adj*_ =.100, *d* = 0.48, and the Bayes factor indicated moderate evidence (*BF*₁₀ = 3.14). The posterior distribution provided strong support for a reduction in the ERP amplitude in the negative condition, compared to the neutral condition *P*(*β* < 0 | *data*) = 0.98, corresponding to an ER of 53.79 (see Fig. 7c).

#### Prospective positivity

For the prospective positivity, there was a main effect of trial type, *F*(2,116) = 12.27, *p* < *.*001, ${\eta}_{p}^{2}$ =.17, with the Bayes factor providing strong evidence for difference across trial types, *BF*₁₀ = 40.76. There was no main effect of affective state condition, and no interaction between condition and trial type, *p*s >.618, *BFs*₁₀ < 1 (see Fig. 5b). Pairwise analyses revealed that There was no reliable difference between ongoing task target trials and ongoing task non-target trials, *t*(116) = 1.56 *p*_*adj*_ =.362, *d* = 0.23, with the Bayes factor indicating only anecdotal evidence for a difference (*BF*₁₀ = 1.09). In contrast, prospective positivity amplitude for PM trials was higher than for ongoing task non-target trials, *t*(116) = 4.85, *p*_*adj*_ <.001, with extreme Bayesian evidence supporting the effect (*BF*₁₀ > 100). Similarly, PM trials also elicited higher amplitudes than ongoing task target trials, *t*(116) = 3.29, *p*_*adj*_ =.004, *d* = 0.34, *BF*₁₀ = 50.

To summarize, the analysis of the electrophysiological data revealed significant interactions between affective states and trial types for the N300 and FN400 components, indicating that the relationship between affective state and ERP amplitude differed across trial types. However, follow-up comparisons did not survive correction for multiple testing. Consequently, the descriptive pattern observed for PM trials should be interpreted cautiously and not as evidence for reliable differences between affective state conditions. No comparable interaction was observed for the P3b or prospective positivity components.

### Intrusive thoughts

**Fig. 6:**
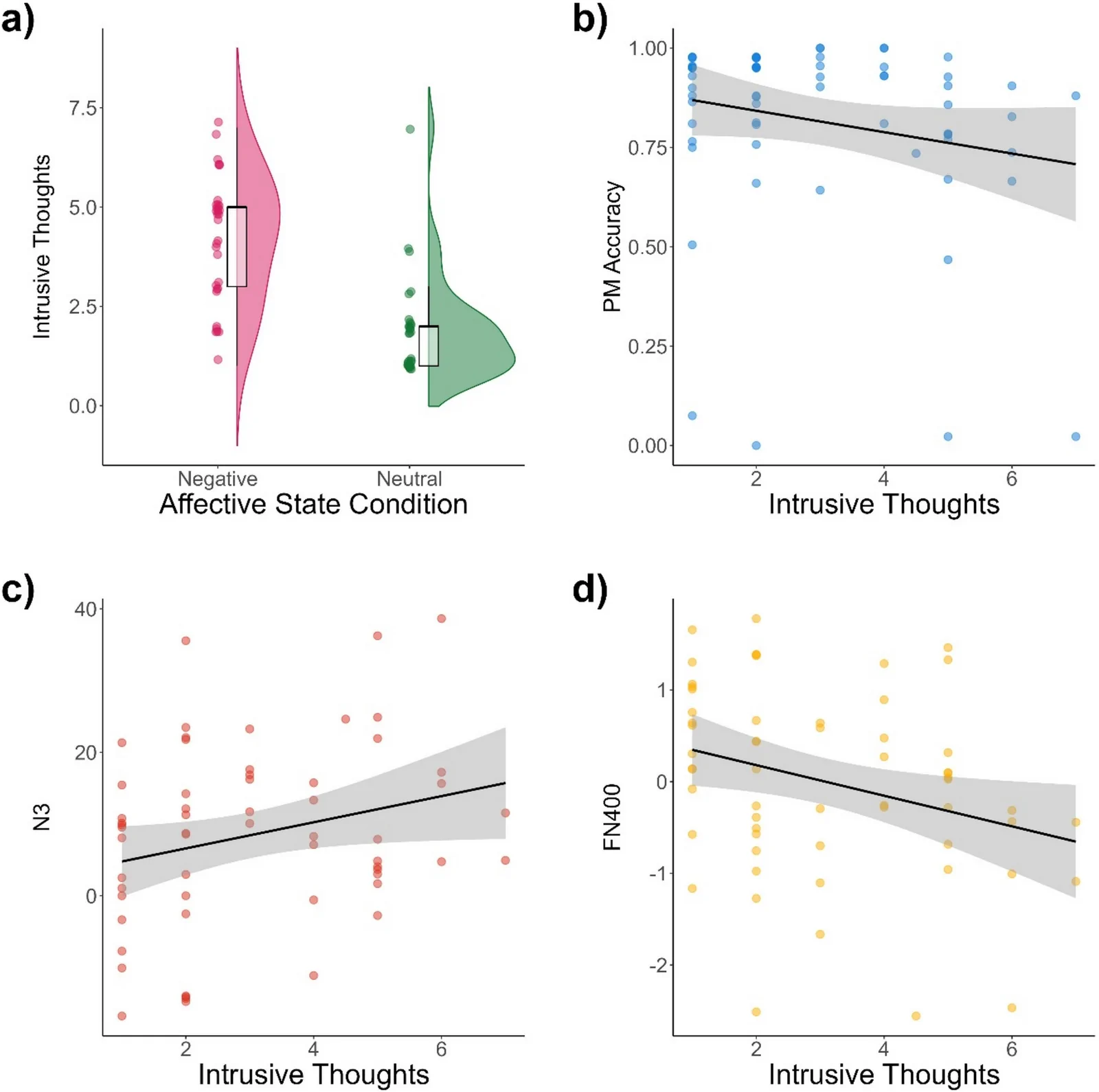
Intrusive thoughts, PM accuracy and ERP components. *Note.* The figure shows (**a**) the amount of reported intrusive thoughts as a function of affective state condition, and the correlations between intrusive thoughts and (**b**) PM accuracy, (**c**) the N300 component, and (**d**) the FN400 component

**Fig. 7:**
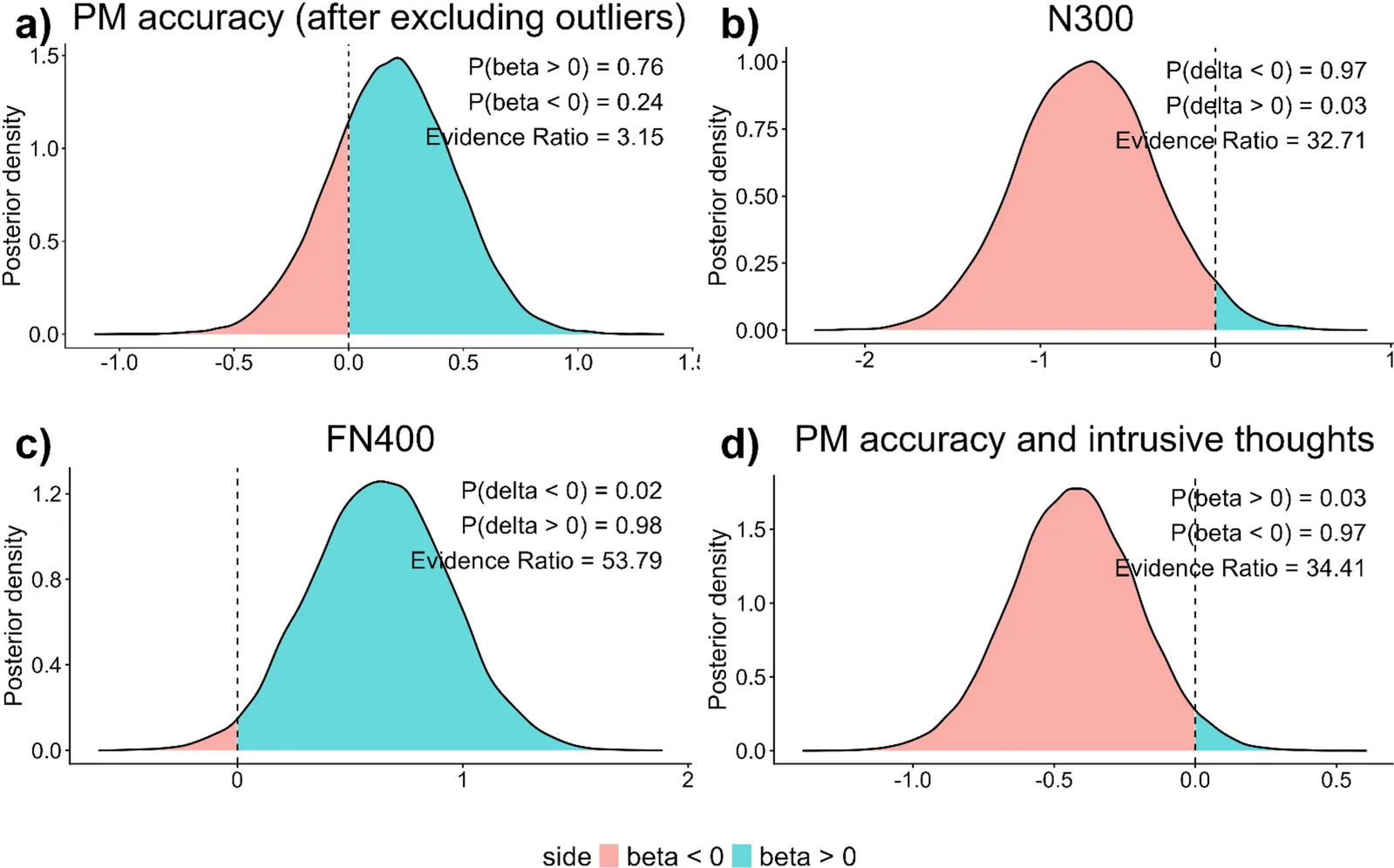
Posterior distributions and evidence ratio for estimated effects and contrasts. Note. Shaded areas represent posterior probability mass below (red) and above (blue) zero. The dashed vertical line denotes the null value (0). Values reported in each panel indicate the posterior probabilities associated with each direction of the effect and the corresponding evidence ratio (ER), which quantifies the relative posterior support for one direction over the opposite direction. In panels (**a**), (**b**), and (**d**), β denotes the coefficient for the effect of affective state condition, coded as negative =  − 1, and neutral =  + 1; therefore, positive β values indicate higher outcome values in the neutral relative to the negative affect condition, whereas negative β values indicate higher outcome values in the negative relative to the neutral mood condition. In panels (**b**) and (**c**), δ denotes planned contrasts comparing the neutral and negative mood conditions within PM trials. Positive δ values indicate larger amplitudes in the neutral relative to the negative mood condition, whereas negative δ values indicate larger amplitudes in the negative relative to the neutral mood condition. For the N300, a negative-going component, more negative amplitudes correspond to larger component amplitudes

Additional analyses were run to explore the relationship between intrusive thoughts and behavioral and ERP components of PM. As intrusive thoughts were not normally distributed in our sample, we reported the results of non-parametric statistical tests. A Mann–Whitney test revealed that participants in the negative condition reported thinking about the film clips while working on the cognitive tasks (*Mdn* = 5) significantly more often than participants in the neutral condition (*Mdn* = 2), *U* = 89.5, *p* <.001, *r* = 0.70, *BF*₁₀ > 100. In addition, there was a statistically significant negative relationship between intrusive thoughts and PM performance, *χ*^*2*^(1) = 5.07, *p* =.024. However, the Bayes factor indicated anecdotal evidence for the presence of an association (BF₁₀ = 2.78), suggesting that the overall evidence for the effect was limited. Nevertheless, the posterior distribution provided strong support for a negative relationship, *P*(*β* < 0 | *data*) = 0.97, corresponding to an ER of 34.41, indicating stronger support for a negative than a positive association (see Fig. 7d). To check whether intrusive thoughts were related to ERP components, we conducted four exploratory bootstrap correlations between intrusive thoughts and components (see Fig. 6). There was a significant correlation between N300 amplitude and intrusive thoughts, *r* =.32, *p* =.012, with the Bayes factor indicating moderate evidence (*BF*₁₀ = 3.99), indicating that higher levels of intrusive thoughts were associated with smaller (i.e., less negative) N300 amplitudes. The correlation between intrusive thoughts and FN400, P3b, and prospective positivity were not supported (FN400: *r* = −.19, *p* =.151, *BF*₁₀ = 0.96; P3b: *r* = −.04, *p* =.778, *BF*₁₀ = 0.33; Prospective positivity = *r* = −.01, *p* =.960, *BF*₁₀ = 0.43.) (Fig. 7).

## Discussion

The present study investigated for the first time the effects of negative affective state on the neural correlates of PM to test the predictions of resource capacity and affect-as-information models of emotion-cognition interactions. Previous contrasting findings (Kliegel et al., 2005; Pupillo et al., 2021; Rummel et al., 2012; Schnitzspahn et al., 2014) had raised questions about the nature of the influence of negative affective state on PM. We addressed these questions by investigating different neural processes underlying PM indexed by several ERP components under negative in comparison with neutral affective state. Results showed that the negative affective state was detrimental to ongoing task performance and interacted with some of the earlier neural correlates of PM performance. In particular, significant interactions between affective state and trial type were observed for the N300 and FN400 components, indicating that the relationship between affective state and ERP amplitude depended on trial type. However, the follow-up comparisons did not survive correction for multiple comparisons. Consequently, the nature of these interactions should be interpreted cautiously, and the descriptive patterns should not be taken as evidence for specific differences between affective state conditions within specific trial types. By contrast, no effect was observed in the components associated with the detection of low-probability targets and intention execution (P3b and prospective positivity, respectively). Additional analyses showed that intrusive thoughts were higher in the negative than neutral conditions. Intrusive thought levels were negatively associated with PM performance and N300 amplitude. Overall, the pattern appears more consistent with theoretical accounts proposing reduced cognitive resources (and lower neural response) under negative affective states (H. C. Ellis & Ashbrooks, 1988) than with the affect-as-information model which predicts enhanced performance under negative affective states.

### Negative affective state and PM: behavioral and neurophysiological findings

On the behavioral level, although the posterior distribution indicated lower PM performance under negative affective than under neutral affective condition, the overall evidence for a difference between conditions was limited. This finding contrasts with previous studies reporting PM impairments under negative affective states (Pupillo et al., 2021, 2022; Schnitzspahn et al., 2014), and is in line with findings from Niculescu et al. (2024) showing also no difference in PM performance between negative and neutral affective states, and Rummel et al. (2012), showing significant differences between negative and positive affective states, but no difference between negative and neutral conditions. On the contrary, negative mood influenced ongoing task performance, with participants performing better in the neutral condition compared to the negative one. This was only for the blocks where participants performed PM and ongoing tasks simultaneously, suggesting that negative mood was particularly problematic where processing capacity was stretched. These findings are in line with studies showing impaired working memory performance under negative affective states (Figueira et al., 2017; Li et al., 2012; Spies et al., 1996).

Using ERPs, we aimed to clarify the effect of negative affective states on prospective remembering, and more precisely to differentiate between cue detection and retrieval processes. Although the difference between negative and neutral affective states in the N300 and FN400 amplitudes did not survive correction for multiple comparisons, the overall pattern of results was consistent with the possibility that affective state varied as a function of trial type. Bayes factors provided only anecdotal-to-moderate evidence for nonzero effects, whereas the posterior distributions showed substantially greater support for reductions in ERP amplitudes under negative relative to neutral affective states. It is worth noting that, although the present sample size was based on previous ERP studies, the effect of affective states may be smaller than our study was equipped to detect**.** Nevertheless, these findings provide the foundation for future investigations with larger samples or more sensitive designs (e.g., within-subject affect manipulations) to clarify the nature and magnitude of this affect-related modulation in the ERP components related to PM.

The N300 is thought to be associated with PM cue detection in event-based tasks (West, 2011). In line with this notion, the N300 amplitude distinguished PM trials from ongoing task trials. Previous studies have also shown that increased ongoing task load reduced N300 amplitude, suggesting that this ERP component reflects the allocation of attentional resources to monitor for the cues (West, 2011; West et al., 2006). Although the PM-specific contrast did not survive correction for multiple comparisons, the posterior distribution was strongly concentrated in the direction of a larger (more negative) N300 amplitude in the neutral relative to the negative affective state condition. This directional pattern is consistent with the possibility that affective state is associated with differences in N300 amplitude. However, because the corresponding post-hoc comparison did not survive correction for multiple comparisons, this pattern should be interpreted as descriptive rather than as evidence for a reliable difference between conditions.

In line with previous research (Cona et al., 2014; West, 2011), the FN400 was observed over a cluster of mid-frontal electrodes and distinguished PM trials from ongoing task trials. The affective state by trial-type interaction was weaker than for the N300, with only anecdotal evidence from the Bayes factor. Nevertheless, the PM contrast showed a consistent directional tendency, with the posterior distribution providing strong support for a reduction in FN400 amplitude under negative compared to neutral affective states. The FN400 was also found in studies investigating the neural correlates of recognition memory (Curran, 2000; J. M. Jennings & Jacoby, 1993), and thus it has been suggested to reflect the automatic recognition of PM cues which is thought to support activation of the intention (Cona et al., 2014).

No affect-related influence was observed in the P3b and the prospective positivity amplitudes. Further, the P3b amplitude failed to distinguish ongoing task trials from PM trials. This is consistent with studies suggesting that the P3b is more pronounced for perceptually distinctive or salient PM cues (e.g., Donchin & Fabiani, 1991). For example, studies manipulating the emotional valence and therefore the salience of the cues have typically reported an increased P3b (e.g., Cona et al., 2014). In the present study, the PM cues were not more salient than the ongoing task stimuli or perceptually different from them. This might explain why the P3b amplitude for the PM cues was similar to the amplitude elicited from the ongoing task target trials. Other studies using resource-demanding ongoing tasks also found no differences between the P3b elicited by PM and ongoing task trials and suggested that the prospective positivity may better distinguish PM trials from ongoing task trials (West, Wymbs, Jakubek, & Herndon, 2003). In line with these studies, in the present study the prospective positivity increased for PM cues compared to ongoing task trials. However, the prospective positivity was not influenced by negative affective state.

### Linking behavior and ERP within the resource-allocation model

Taken together, the ERP findings provide tentative support for the resource-allocation model, which predicts that affective demands draw on limited processing capacity. This result is in line with studies finding decreased amplitude under negative affective states in ERP components related to resource allocation in an oddball task (e.g., Meinhardt & Pekrun, 2003). The observed pattern is consistent with the possibility that negative affective states draw modest resources away from orienting of attention toward PM cues (N300) and familiarity-based cue recognition (FN400), whereas later, more controlled post-detection stages, such as switching from the ongoing task to retrieval of the PM intention, indexed by the prospective positivity, were not influenced by negative affective state.

Despite the directional tendency toward poorer PM performance under negative affective states, overall evidence for a behavioral difference remained limited. This may be due to the relative ease of the task, reflected in participants’ high average accuracy. We intentionally used a low-demand PM task to maximize overall PM accuracy, thus increasing the number of trials in our ERP analysis. However, this decision likely reduced the sensitivity to detect behavioral differences due to the near-ceiling performance. Beyond this methodological explanation, the dissociation between neurophysiological modulation and preserved PM accuracy may reflect the robustness of PM performance under low task demands. According to the multiprocess framework of PM (McDaniel & Einstein, 2000), successful performance might rely on both strategic and automatic retrieval processes. In the present paradigm, the relatively simple PM task might have allowed participants to rely predominantly on automatic retrieval processes, which might be less susceptible to transient affective influence. Moreover, repeated exposure to the same cues might have strengthened cue-intention associations, further facilitating spontaneous retrieval (Einstein et al., 2005a, 2005b). Negative affective states might have interfered with processes involved in the detection and recognition of the cues, but this interference was apparently insufficient to impact behavioral performance. Future studies should examine whether PM tasks requiring stronger monitoring to detect the PM cues might be more susceptible to negative affective state influences, and how this might influence both early and late ERP components.

Although overall ongoing task performance improved in the PM blocks, participants assigned to the negative affective state condition performed worse than their neutral counterparts. The general improvement following the introduction of the PM task may reflect a practice effect, which could have masked PM-related interference (see, e.g., Cona et al., 2012). Following mood induction, participants in the negative affective state condition showed impaired ongoing task performance in the PM blocks relative to the neutral condition. This finding is consistent with a recent PM study reporting poorer ongoing task performance in PM blocks after participants viewed negative video content (Vicentin et al., 2023). These patterns suggest that the negative affective state may have interfered with cognitive processes underlying the execution of the 2-back task.

The reason for detecting the influence of affective state on ongoing task performance, but not on PM performance, might be also attributable to the higher number of trials of the ongoing task (404 trials) compared to the PM task (42 trials). Future studies could consider increasing the number of PM trials to improve statistical power for detecting behavioral differences.

In the current study, we focused on electrophysiological components related to PM retrieval, rather than on components which may characterize working memory processes. As a result, we did not analyze important components known to support n-back performance, such as the P2 and the N2, which are associated with updating (Lenartowicz et al., 2010) and inhibitory control (W. Wang et al., 2021), respectively. Another working memory related ERP, the contralateral delay activity (CDA), which reflects the maintenance of representations, has been shown to be influenced by negative affective states in a change detection task by Figueira et al., 2017. Future studies could explore in more detail which ERP components are sensitive to mood effects on n-back performance to better understand the processes involved.

### Correlations between intrusive thoughts and ERP components

The resource-allocation model predicts that the detrimental influence of affective states is explained by increased intrusive thoughts. Accordingly, the present study investigated the amount of intrusive thoughts that participants experienced during the PM tasks. Results showed that participants in the negative mood condition reported substantially more intrusive thoughts related to the film clips while doing the PM task compared to participants in the neutral mood condition. This result is in line with previous studies showing increased intrusive thoughts under negative affective states (Smallwood et al., 2009). In addition, results from the present study provided tentative evidence that intrusive thoughts were associated with poorer PM performance, in line with findings from Niculescu et al. (2024).

Intrusive thoughts were also associated with reduced N300 amplitudes, suggesting that cue-detection processes may be particularly vulnerable to interference from task-unrelated thoughts. In line with the resource-allocation model (H. C. Ellis & Ashbrooks, 1988), the increase in intrusive thoughts experienced by participants in the negative affective state condition might have drawn upon limited cognitive resources and resulted in reduced electrophysiological responses. By contrast, the FN400 amplitude showed no reliable association with intrusive thoughts, suggesting that familiarity-based recognition processes may be less affected by the detrimental effects of task-unrelated thoughts. Once the cue is detected, intention retrieval might be relatively automatic (Cona et al., 2014) and be more resistant to the transient interference caused by intrusive thoughts.

### Limitations and future directions

More research is needed to clarify if the affect-related reduction in the early ERP components of PM can be generalized or is rather task-specific. Although we ensured that the images serving as PM cues were not more salient or distinctive than ongoing task stimuli, the PM cues in the present study (i.e., specific pictures) were processed as part of the ongoing task (i.e., comparing each picture with the one shown two trials earlier). According to the PM literature (McDaniel & Einstein, 2000, 2007), the detection of such so-called focal cues requires less controlled attention than non-focal tasks (i.e., in which the cues are not processed as part of the ongoing task). This low cognitive demand is reflected in participants’ relatively high average accuracy in the PM and the ongoing task. Thus, a low-effort retrieval might explain why negative affective state influenced the early ERP components linked to the detection and recognition of the cue but did not translate into PM deficits.

It is important to note that the specific affective state examined in this study was elicited through a mood induction procedure, which may have influenced the observed results. While this procedure is considered effective in inducing negative affective states (Fernández-Aguilar et al., 2018), the generated affective states are likely to be perceived by participants as external to the task. This is a crucial aspect, as it has been suggested that incidental, task-unrelated may impact cognition differently than task-related emotions (Inzlicht et al., 2015). Specifically, task-related emotions, such as those triggered by conflict within a task (e.g., incongruent stimuli in a Stroop task; Dreisbach & Fischer, 2012), can signal the need for greater cognitive control, ultimately enhancing performance (Dignath et al., 2020). This effect may be particularly beneficial for PM, where resolving the conflict between retrieving an intention and continuing to perform the ongoing task is essential. Future research could explore whether task-related negative emotions influence PM differently from incidental negative affective state.

In the present study, we measured affective states several times, to capture possible fluctuations in affective states. However, because of the natural decay of induced mood (Kliegel et al., 2005; Schnitzspahn et al., 2014), it is possible that affective states may have fluctuated even within individual blocks. To better track moment-to-moment changes, future research should consider more frequent mood assessments, allowing for a clearer link between momentary affective states and the likelihood of remembering the intention. For instance, Blain and Rutledge (2020) had participants rate their affective state every three to four trials, reaching almost trial-level measurement of affective state.

An important limitation of the present study concerns the dissociation of affective dimensions. Because the mood induction procedure simultaneously influenced both valence and arousal, the negative condition was characterized by lower valence and higher arousal compared to the neutral condition. As a result, we cannot conclusively determine whether the observed behavioral and electrophysiological effects are driven specifically by valence, by arousal, or by an interaction between these dimensions. Consequently, the present findings should be interpreted as reflecting the effects of negative affective states more broadly, rather than isolating the contribution of a single affective dimension. Future research should aim to disentangle these effects by orthogonally manipulating valence and arousal, for example, by selecting stimuli that vary independently along these dimensions or by including both high- and low-arousal conditions within each valence category.

It should be noted that in the present study we investigated the influence of negative affective states on pre-determined ERP components and time windows, a methodological approach which is very common in the ERP literature and allows for clear conclusions on the underlying cognitive mechanisms involved. However, these approaches have the disadvantage of focusing attention on specific components and time windows, instead of the whole data space. One recent approach, the mass univariate approach (Pernet et al., 2015), allows simultaneous testing on all electrodes and all timepoints, broadening the investigation of potential effects. Multivariate approaches, such as information decoding via a timepoint-by-timepoint classifier across electrode space, may be less suitable—while they offer a more powerful way of analyzing EEG data (Rozman et al., 2024), the classifier performance is limited by the ratio of trials to number of channels, meaning that the number of PM trials that could be obtained from an experiment would need to be considerably larger to ensure sufficient data are available. Future investigations could use these approaches to identify sensory representations of PM cues or emotional representations throughout the task (J. Wang et al., 2024).

It is important to acknowledge that in the present study the negative and neutral stimuli differed not only in the type of emotion elicited but also based on their narrative and semantic properties. Specifically, the negative stimuli consisted of dynamic, narrative-rich film clips, whereas the neutral condition relied on comparatively less dynamic and potentially less semantically dense materials. As a result, the observed effects may not be solely attributable to differences in affective characteristics. Narrative stimuli are more likely to engage deeper semantic processing and self-referential thinking, which may in turn increase the likelihood and persistence of task-unrelated thoughts (Bellana et al., 2022). Consequently, it is possible that the lingering cognitive effects observed following negative mood induction partly reflect differences in narrativity or semantic richness rather than affective states per se. Future research should aim to disentangle these factors by varying affective content and narrative structure systematically and independently, thereby clarifying the extent to which the present findings are driven by emotional versus more general cognitive characteristics of the stimuli.

### Applied implications

The present findings have important applied implications for everyday situations in which prospective memory must be maintained under negative affective states. Many real-world PM demands, such as remembering to ask a question to a colleague during a meeting, or to hand a document to someone when they arrive, occur in contexts in which negative affective states might be involved (e.g., anxiety, worry, rumination). The observed association between negative affect, increased intrusive thoughts, and reduced neural markers of cue detection suggests that emotional states may compromise PM not by impairing intention execution per se, but by disrupting early attentional processes required to notice relevant cues. From an applied perspective, this implies that interventions aimed at supporting PM under negative affect may benefit from targeting cue salience or reducing opportunities for task-unrelated intrusive thoughts. For example, increasing the perceptual distinctiveness of PM cues, externalizing intentions through reminders, or implementing strategies that limit cognitive load may help offset affect-related reductions in attentional resources.

## Conclusions

Overall, the findings of the present study provide tentative support for capacity-based accounts of emotion-cognition interactions. The pattern of affect-related modulation of ERP components, including the association between intrusive thoughts and decreased PM performance, suggests that negative affective states may influence attentional allocation during prospective remembering.

Importantly, Bayesian analyses indicated that, while the evidence for effects should be considered tentative, the posterior distributions consistently favored reductions in the N300 and FN400 amplitudes under negative affective states, pointing to a coherent directional pattern. The present results highlight a potential mechanism through which negative affective states may impact prospective memory, namely via increased task-unrelated thoughts and reduced allocation of attentional resources to cue detection. However, these novel effects were modest and should be interpreted with caution.

More broadly, the present study contributes to the literature by integrating behavioral, electrophysiological, and self-report measures, offering a more nuanced picture of how affective states may influence prospective memory. They also underscore the need for further research into the electrophysiological basis of the detrimental effects of negative mood on prospective memory, to better understand how these effects extend to different PM tasks in particular and cognitive functions more broadly.

## Supplementary Information

Additional file 1.

## Data Availability

The data and scripts supporting the findings of this study are available from the corresponding author upon request.

## Footnotes

1 The choice of the sample size was based on previous studies investigating the effect of cognitive load on PM and related ERPs (e.g., West et al., 2006) and studies exploring affective influences on ERPs (e.g., Meinhardt & Pekrun, 2003). These studies used a sample of 18 and 24 participants, respectively. In the present study, the number of participants within each condition exceeded the number used by the studies described above, as potential exclusions and/or withdrawals were considered.
