# Supplementary material for: The influence of negative affective states on the neurophysiological correlates of prospective memory
Source: Cogn Res Princ Implic. 2026 Aug 9;11:56. doi: 10.1186/s41235-026-00749-3 (PMC13737340; doi:10.1186/s41235-026-00749-3)
Supplement: Supplementary file 1 [file 41235_2026_749_MOESM1_ESM.docx]

**Supplementary Material**

**Further information and pilot data on the film clips used in the mood induction**

As previous research suggests that young and older adults react differently to emotional material (e.g., Schnitzspahn et al., 2014), we asked samples of young and older adults to rate the valence and arousal of partly different film clips in several pilot studies. In total, we conducted three pilot studies in young adults and three pilot studies in older adults until we felt confident that we identified a sufficient number of positive, neutral and negative film clips in each age group. For the present study, we selected the ones that were most effective in inducing the desired emotions (see Table 1 for their duration, valence and arousal ratings from piloting).

The film clips for the young adults were piloted in different samples of 85 participants (age range= 17 – 34, *M*_age_ = 20.29, *SD* = 2.44, 12 male, 73 female), 32 participants ( age range = 19-28, *M*_age_ = 21.44, *SD* = 1.98, 23 female, 9 male) and 21 participants (age range = 18 -25, *M*_age_ = 19.14, *SD* = 1.85, 18 female, 3 male). In the older adults, we used three samples of 9 (age range= 64 – 74, *M*_age_ = 67.78, *SD* = 3.53, 3 male, 6 female), 8 (age range= 64– 79, *M*_age_ = 68.75, *SD* = 5.68, 4 male, 4 female) and 21 (age range= 59– 76, *M*_age_ = 67.42, *SD* = 4.94, 8 male, 11 female) participants, respectively.

**Table S1**.

Duration, Valence and Arousal Scores Obtained in the Pilot Work for the Film Clips Selected for the Main Study

*Note*. Valence and arousal were assessed using a 9-point Likert scales. For valence, low values represent positive mood and high values negative mood. For arousal, low values represent high arousal and high values low arousal.

^a^Scene taken from the motion picture.

^b^Film clip found on You Tube.

| Film clips |  | Condition |  | Duration |  | N |  | Valence (SD) |  | Arousal (SD) |
| --- | --- | --- | --- | --- | --- | --- | --- | --- | --- | --- |
| 1. Cyberbully^a^ |  | Negative |  | 03:36 |  | 85 |  | 7.3 (1.5) |  | 5.3 (2.1) |
| 2. The Green Mile^a^ |  | Negative |  | 03:46 |  | 85 |  | 7.4 (1.4) |  | 5.7 (2.0) |
| 3. The Champ^a^ |  | Negative |  | 02:45 |  | 85 |  | 7.1 (1.7) |  | 6.0 (1.8) |
| 4. Dancer in the Dark^a^ |  | Negative |  | 03:17 |  | 32 |  | 5.4 (1.1) |  | 4.2 (0.9) |
| 5. Beaver documentary^b^ |  | Neutral |  | 03:18 |  | 85 |  | 3.7 (1.4) |  | 5.2 (1.8) |
| 6. Beach images^b^ |  | Neutral |  | 02:20 |  | 32 |  | 3.3 (1.7) |  | 6.9 (1.4) |
| 7. Desert documentary^b^ |  | Neutral |  | 02:41 |  | 21 |  | 4.0 (1.5) |  | 5.0 (1.0) |
| 8. Satellite images^b^ |  | Neutral |  | 03:49 |  | 21 |  | 3.7 (1.3) |  | 7.0 (0.9) |

**Table S2**.

Mean values and standard deviations for the mean amplitudes as a function of trial type and affective state condition

|  |  | Negative Affective State (N = 29) | | |  | Neutral Affective State (N = 28) | | |
| --- | --- | --- | --- | --- | --- | --- | --- | --- |
|  |  | *M* |  | *SD* |  | *M* |  | *SD* |
| *N300^a^* |  |  |  |  |  |  |  |  |
| PM cues |  | 1.023 |  | 4.22 |  | -1.526 |  | 4.22 |
| OT target hits |  | -0.083 |  | 4.08 |  | -1.109 |  | 4.08 |
| OT non-target hits |  | 1.80 |  | 3.79 |  | 0.164 |  | 3.79 |
|  |  |  |  |  |  |  |  |  |
| *P3b^b^* |  |  |  |  |  |  |  |  |
| PM cues |  | 3.69 |  | 2.19 |  | 3.31 |  | 2.42 |
| OT target hits |  | 3.40 |  | 1.77 |  | 3.26 |  | 2.13 |
| OT non-target hits |  | 2.39 |  | 2.39 |  | 2.99 |  | 2.40 |
|  |  |  |  |  |  |  |  |  |
| *FN400^c^* |  |  |  |  |  |  |  |  |
| PM cues |  | -1.06 |  | 2.51 |  | 0.56 |  | 2.54 |
| OT target hits |  | -1.00 |  | 2.45 |  | -0.80 |  | 2.46 |
| OT non-target hits |  | -2.21 |  | 1.81 |  | -1.50 |  | 2.20 |
|  |  |  |  |  |  |  |  |  |
| *Prospective positivity^d^* |  |  |  |  |  |  |  |  |
| PM cues |  | 3.53 |  | 3.62 |  | 2.32 |  | 2.04 |
| OT target hits |  | 3.70 |  | 2.60 |  | 2.62 |  | 1.84 |
| OT non-target hits |  | 1.75 |  | 2.10 |  | 2.38 |  | 1.58 |
| ^a^N300 peak amplitude was clustered across electrodes O1, Oz, and O2.  ^b^P3b peak amplitude was clustered across electrodes POz, P1, CPz, CP2, and Pz  ^c^ FN400 mean amplitude was clustered across electrodes FCz, F1, AFz, F2, and Fz  ^d^ Prospective positivity mean amplitude was clustered across electrodes Pz, CP1, Cz, CP2, and CpZ. | | | | | | | | |

***Table S3***

*Results of the analysis of the mean amplitudes.*

|  |  | *F* | *df* | *p* | $\boldsymbol{ƞ}_{\boldsymbol{p}}^{\boldsymbol{2}}$ (95% CI) |
| --- | --- | --- | --- | --- | --- |
| *N300^a^* |  |  |  |  |  |
| Affective state condition |  | 3.97 | 1, 58 | .094 | .05 [.00, .16] |
| Trial type |  | 17.74 | 2, 116 | **< .001** | .26 [.13, .38] |
| Trial type x Mood |  | 3.78 | 2, 116 | **.018** | .07 [.00, .16] |
|  |  |  |  |  |  |
| *P3b^b^* |  |  |  |  |  |
| Affective state condition |  | 0.63 | 1,58 | .632 | .00 [.00, .09] |
| Trial type |  | 7.66 | 2,116 | **< .001** | .12 [.02, .23] |
| Trial type x Mood |  | 0.48 | 2,116 | .618 | .00 [.00, .06] |
|  |  |  |  |  |  |
| *FN400^c^* |  |  |  |  |  |
| Affective state condition |  | 0.95 | 1,58 | .333 | .02 [.00, .13] |
| Trial type |  | 49.27 | 2,116 | **< .001** | .46 [.33, .56] |
| Trial type x Mood |  | 3.80 | 2,116 | **.034** | .06 [.00, .15] |
|  |  |  |  |  |  |
| *Prospective Positivity^d^* |  |  |  |  |  |
| Affective state condition |  | 0.23 | 1,58 | .632 | .00 [.00, .10] |
| Trial type |  | 7.66 | 2,116 | **< .001** | .12 [.06, .29] |
| Trial type x Mood |  | 0.48 | 2,116 | .618 | .00 [.00, .04] |
| ^a^N300 peak amplitude was clustered across electrodes O1, Oz, and O2.  ^b^P3b peak amplitude was clustered across electrodes POz, P1, CPz, CP2, and Pz  ^c^ FN400 mean amplitude was clustered across electrodes FCz, F1, AFz, F2, and Fz  ^d^ Prospective positivity mean amplitude was clustered across electrodes Pz, CP1, Cz, CP2, and CpZ. | | | | | |

**Matlab script for removing artifacts**

%-----------------------------------------------------------------------%

%-----------------------------------------------------------------------%

% define the path to the data

filepath = [];

% Define the subjects

Subjects = 1:n;

% loop over the subjects

for ns = 1: numel(Subjects,)

% get the EEG file

EEG = pop_loadset(Subjects(nd) filepath);

%sort out the eye channels locations

EEG=pop_chanedit(EEG, 'changefield',{65 'theta' '-25'},'changefield',{65 'radius' '0.58333'},'changefield',{65 'X' '0.87543'},'changefield',{65 'Y' '0.40822'},'changefield',{65 'Z' '-0.25882'},'changefield',{65 'sph_theta' '25'},'changefield',{65 'sph_phi' '-15'});

EEG=pop_chanedit(EEG, 'changefield',{66 'theta' '-27'},'changefield',{66 'radius' '0.69444'},'changefield',{66 'X' '0.72987'},'changefield',{66 'Y' '0.37189'},'changefield',{66 'Z' '-0.57358'},'changefield',{66 'sph_theta' '27'},'changefield',{66 'sph_phi' '-35'});

EEG=pop_chanedit(EEG, 'changefield',{67 'theta' '-42'},'changefield',{67 'radius' '0.65556'},'changefield',{67 'X' '0.65616'},'changefield',{67 'Y' '0.59081'},'changefield',{67 'Z' '-0.46947'},'changefield',{67 'sph_theta' '42'},'changefield',{67 'sph_phi' '-28'});

EEG=pop_chanedit(EEG, 'changefield',{68 'theta' '42'},'changefield',{68 'radius' '0.65556'},'changefield',{68 'X' '0.65616'},'changefield',{68 'Y' '-0.59081'},'changefield',{68 'Z' '-0.46947'},'changefield',{68 'sph_theta' '-42'},'changefield',{68 'sph_phi' '-28'});

EEG=pop_chanedit(EEG, 'changefield',{69 'theta' '-108'},'changefield',{69 'radius' '0.639'},'changefield',{69 'X' '-0.28'},'changefield',{69 'Y' '0.862'},'changefield',{69 'Z' '-0.423'},'changefield',{69 'sph_theta' '108'},'changefield',{69 'sph_phi' '-25'});

EEG=pop_chanedit(EEG, 'changefield',{70 'theta' '108'},'changefield',{70 'radius' '0.639'},'changefield',{70 'X' '-0.28'},'changefield',{70 'Y' '-0.862'},'changefield',{70 'Z' '-0.423'},'changefield',{70 'sph_theta' '-108'},'changefield',{70 'sph_phi' '-25'});

```
%re-reference
EEG = pop_reref(EEG,[],'refstate',0,'exclude',[65:EEG.nbchan]); % Average Reference

% low-pass filter
EEG = pop_firws(EEG, 'fcutoff', 40, 'ftype', 'lowpass', 'wtype', 'kaiser', 'warg', 5.65326, 'forder', 184, 'minphase', 0);
	%---------------------------------------------------------------%
	% run FASTER
	%---------------------------------------------------------------%
	%take out the 32 channels from EEG data to feed into faster
backupEEG   = EEG.data;           % back-up full data matrix
backupNumchan = EEG.nbchan;
backuplocs  = EEG.chanlocs;

% keep only the 64 scalp channels for FASTER
EEG.data    = EEG.data(1:64,:,:);
EEG.nbchan  = 64;
EEG.chanlocs = EEG.chanlocs(1,1:64);

% --- FASTER epoch-level statistics and thresholding ---
epoch_props = epoch_properties(EEG,1:64);  	     % compute epoch properties
epochs_threshold = min_z(epoch_props);           % z-score thresholding (default ±3 SD)
bad_epochs = find(epochs_threshold);             % indices of epochs to reject

     %now restructure my EEG data to also include external channels
     EEG.data=[EEG.data; backupEEG(65:backupNumchan,:,:)];
     EEG.nbchan=backupNumchan;
     EEG.chanlocs=backuplocs;

     %reject the bad epochs
     EEG = pop_rejepoch( EEG, bad_epochs,0);
```

%---------------------------------------------------------------%

% now run ICA %

%---------------------------------------------------------------%

EEG = pop_runica(EEG, 'icatype','runica','dataset',1,'options',{'extended' 1},'chanind',[1:70] );

% automatically detect artifactual components

[art, horiz, vert, blink, disc,soglia_DV, diff_var, soglia_K, meanK, soglia_SED, SED, soglia_SAD, SAD,

soglia_GDSF, GDSF, soglia_V, nuovaV]=ADJUST(EEG,reportname);

% take record of these components

fprintf(fid5,'%s\t %i\n','HEM_components',length(horiz));

fprintf(fid5,'%s\t %i\n','VEM_components',length(vert));

fprintf(fid5,'%s\t %i\n','blink_components',length(blink));

fprintf(fid5,'%s\t %i\n','discontinuity_components',length(disc));

%subtract them from the data

EEG = pop_subcomp( EEG, art, 0);

%---------------------------------------------------------------%

% Blink and eye movement removal

%---------------------------------------------------------------%

% detrend eye channels only

backupEEG=EEG.data; %store data in a variable as a backup

EEG = eegF_Detrend(EEG);

detrendEEG=EEG.data;

EEG.data=[backupEEG(1:64,:,:); detrendEEG(65:70,:,:)];

% now remove trials with blinks

backupEEG=EEG.data; %store data in a variable as a backup

```
% make HEOG channel
EEG.chanlocs(67).labels='HEOG';
EEG.data(67,:,:)=EEG.data(67,:,:)-EEG.data(68,:,:);
[EEG,Trials2Remove2] = SCADS_RemoveEyeMovements(EEG,[65,67],[blinkstart eyemovend],25,1);
% reinsert the upper eye channel
EEG.data(65,:,:)=backupEEG(65,:,:);
EEG.data(67,:,:)=backupEEG(67,:,:);

EEG=pop_select(EEG,'notrial',Trials2Remove1);
EEG=pop_select(EEG,'notrial',Trials2Remove2);
% take stock of all the rejected epochs
fprintf(fid5,'%s\t %i\n','blinks',length(Trials2Remove1));
%take stock of all the rejected epochs
fprintf(fid5,'%s\t %i\n','eye movs',length(Trials2Remove2));


EEG = pop_saveset( EEG, [subjects{ns} '_ALL_rejected.set'], filepath1);

     fclose(fid5);

end; % end of the subjects’loop
```

**References**

Faul, F., Erdfelder, E., Buchner, A., & Lang, A.-G. (2009). Statistical power analyses using G*Power 3.1: Tests for correlation and regression analyses. Behavior Research Methods, 41(4), 1149–1160. <https://doi.org/10.3758/BRM.41.4.1149>

Schnitzspahn, K. M., Thorley, C., Phillips, L., Voigt, B., Threadgold, E., Hammond, E. R., Mustafa, B., & Kliegel, M. (2014). Mood impairs time-based prospective memory in young but not older adults: The mediating role of attentional control. Psychology and Aging, 29(2), 264–270. https://doi.org/10.1037/a0036389
